# Synthesis of Highly Concentrated Silver Nanoparticle Dispersions and Their Integration into Cellulose Films for Catalytic and Antibacterial Applications

**DOI:** 10.1021/acsomega.5c13300

**Published:** 2026-07-22

**Authors:** Rodolfo P. P. Nunes, Vitor G. Vital, Nubia G. dos Santos, Suzan Pantaroto de Vasconcellos, Larissa V. F. Oliveira, Fernanda F. Camilo

**Affiliations:** † Chemistry Department, Institute of Environmental, Chemical and Pharmaceutical Sciences, 28105 Federal University of São Paulo, Diadema, São Paulo 09913-030, Brazil; ‡ Center of Natural Sciences and Humanities, 74362 Federal University of ABC, Santo André, São Paulo 09210-580, Brazil

## Abstract

Silver nanoparticles (AgNPs) have attracted considerable attention due to their outstanding catalytic and antimicrobial properties. Nevertheless, most aqueous syntheses yield colloids with low metal concentrations and limited long-term stability, restricting their practical use. To address these challenges, highly concentrated and stable AgNP dispersions were prepared, reaching concentrations of up to 100 mM, by chemical reduction of AgBF_4_ with tetrabutylammonium borohydride in the presence of polyvinylpyrrolidone (PVP) as a stabilizer. The dispersions contained predominantly spherical nanoparticles with narrow size distributions and remained stable for at least 1 month. For improved reusability, the AgNPs were incorporated into regenerated cellulose films produced in 1-butyl-3-methylimidazolium chloride. Owing to its abundance, renewability, and biocompatibility, cellulose provides a sustainable and environmentally friendly platform for supporting metal nanoparticles, offering easy handling and potential for reuse. The resulting AgNP-cellulose composites contained up to 5 wt % of silver and preserved the cellulose type II structure. Structural, thermal, and morphological analyses confirmed the homogeneous distribution of PVP-stabilized AgNPs within the matrix. The hybrid films exhibited high catalytic activity toward 4-nitrophenol reduction and could be reused for at least five consecutive cycles with minimal loss of performance. In addition, antibacterial assays against *Escherichia coli* (ATCC 8739) and *Staphylococcus aureus* (ATCC 25923) showed antibacterial activity, with a statistically supported and concentration-dependent response that was more pronounced for the Gram-positive strain. This work demonstrates a straightforward strategy to combine concentrated AgNP dispersions and cellulose supports, yielding recyclable catalytic and antimicrobial materials.

## Graphical abstract

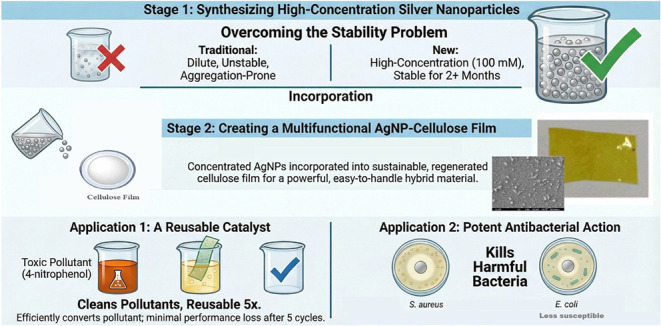

## 1 Introduction

Metallic nanomaterials—including gold (Au), silver (Ag), platinum (Pt), and palladium (Pd)—exhibit unique physicochemical properties at the nanoscale that are not observed in their bulk forms.^1^ Among these, silver nanoparticles (AgNPs) stand out as a cost-effective alternative to Au, Pt, and Pd, offering broad-spectrum antimicrobial activity and high catalytic efficiency in a wide range of reactions.^2–9^ These properties have attracted considerable attention for applications in catalysis, electronic devices, and antimicrobial coatings, thereby stimulating the development of synthetic methods for AgNP production.^10^

Conventional bottom-up routes, however, often yield colloids with relatively modest silver concentrations.^11–15^ More concentrated AgNP dispersions are therefore desirable, particularly because they may facilitate incorporation into coatings and composite materials without additional concentration steps that can promote aggregation and loss of functionality.^16^ In response, several strategies have been proposed to obtain more concentrated and stable aqueous AgNP dispersions. In this context, Anderson et al. reported the preparation of AgNP dispersions with concentrations above 1 mM (0.11 g L^–1^) using comb-graft copolymers derived from poly­(ethylene glycol) (PEG) acrylate/maleic anhydride.^17^ Shahzad and coworkers later developed rapid aqueous-phase methods employing multifunctional polymers and branched polyethylenimine (PEI), achieving highly concentrated AgNP dispersions, in some cases up to 100 mM or higher, while also demonstrating catalytic activity in the NaBH_4_-mediated reduction of 4-nitrophenol.^18,19^ Francesko et al. subsequently described an eco-friendly one-step sonochemical synthesis yielding concentrated AgNPs stabilized by chitosan (4.7 mM, 0.51 g L^–1^), in which chitosan acted simultaneously as reducing and stabilizing agent. The resulting AgNP–chitosan hybrids showed antibacterial activity against *E. coli* and *S. aureus*, highlighting their potential for biomedical and antibiofilm applications.^20^

Although AgNP dispersions in the 1–100 mM range have been reported and are generally regarded as high-concentration systems, concentration alone is no longer sufficient to establish the significance of a new AgNP system. Additional aspects such as colloidal stability, particle-size control, and preservation of functionality in downstream applications have become equally important. Furthermore, even well-stabilized colloids remain thermodynamically prone to aggregation over time, which may compromise catalytic and antimicrobial performance. This limitation becomes particularly relevant in applications where facile recovery, reduced leaching, and reusability are required, such as in heterogeneous catalysis and long-term antimicrobial materials.

In this regard, immobilization of AgNPs onto solid supports has emerged as a promising strategy to improve stability, reduce nanoparticle loss, and enable recovery and sustained use. Cellulose films represent an attractive platform for this purpose because they are nontoxic, water-insoluble, and derived from an abundant renewable biopolymer. In addition, their hydroxyl-rich structure can favor interactions with silver species, which may help minimize leaching and facilitate reuse.^21–24^ Nevertheless, some AgNP–cellulose systems reported to date rely on chemically modified cellulose or other derivatized biopolymers, which partly undermines the advantages of using a naturally abundant and renewable support by introducing additional synthetic steps and chemical reagents.^25,26^ Moreover, cellulose in powdered or fibrous form can complicate separation, handling, and reuse in practical systems, thereby limiting its applicability in scalable and sustainable processes.^27–29^

Therefore, although high-concentration AgNP dispersions and AgNP–cellulose materials have both been reported, systems combining concentrated aqueous AgNP dispersions with *ex situ* immobilization onto practical film-forming supports prepared from nonderivatized cellulose have received less attention, especially when catalytic performance, antibacterial activity, and reusability are evaluated together. In this context, the contribution of the present work is not based on concentration alone, but on demonstrating a straightforward strategy that couples a stable silver tetrafluoroborate, tetrabutylammonium borohydride, and polyvinylpyrrolidone (AgBF_4_/TBABH_4_/PVP) colloidal system with regenerated cellulose films prepared from unmodified cellulose, yielding renewable multifunctional materials for catalytic and antibacterial applications.

## 2 Experimental Part

### 2.1 Preparation of Highly Concentrated Aqueous Dispersions of Silver Nanoparticles

To prepare the aqueous silver nanoparticle dispersions, a 10% w/w polyvinylpyrrolidone (PVP) solution was first prepared by dissolving 10.00 g of PVP (Mw = 10,000 g·mol^–1^, Aldrich) in 90.00 g of distilled water. This solution, referred to as PVP_10%, was stirred at room temperature for 24 h. In a separate glass vessel, 10.00 mL of the PVP_10% solution was mixed with varying amounts of silver tetrafluoroborate (AgBF_4_, 98%, Aldrich) to obtain final silver salt concentrations of 5 mM, 10 mM, 25 mM, 50 mM, and 100 mM. The dissolution of AgBF_4_ was carried out under magnetic stirring at room temperature. In parallel, weighed amounts of tetrabutylammonium borohydride (TBABH_4_, 98%, Aldrich) were dissolved in 2.0 mL of the PVP_10% solution. The mass of TBABH_4_ was calculated to maintain a 1:1 molar ratio with Ag^+^ in each case. After, the TBABH_4_ solution was added dropwise—approximately one drop per second—to the AgBF_4_ solution under vigorous stirring. After the addition was complete, the reaction mixture was stirred at room temperature for an additional 30 min. The resulting dispersions were denoted as XmM_AgNP, where X refers to the nominal silver salt concentration. The specific reagent quantities used in each synthesis are summarized in Table 1.

**Table 1:** Experimental Parameters Used for the Synthesis of AgNP Dispersions at Different Nominal Ag^+^ Concentrations

Sample ID	Mass of AgBF_4_ (mg)	Amount of AgBF_4_ (mmol)	Amount of TBABH_4_ (mmol)^a^	Concentration of TBABH_4_ solution (mmol·L^–1^)	Nominal Ag^+^ concentration (mM)
5 mM_AgNP	11.6	6.00 × 10^–2^	6.00 × 10^–2^	30.0	5
10 mM_AgNP	23.3	12.0 × 10^–2^	12.0 × 10^–2^	60.0	10
25 mM_AgNP	58.4	30.0 × 10^–2^	30.0 × 10^–2^	150	25
50 mM_AgNP	116.8	60.0 × 10^–2^	60.0 × 10^–2^	300	50
100 mM_AgNP	233.6	120 × 10^–2^	120 × 10^–2^	600	100

a Dissolved in 2.00 mL of the PVP 10% solution.

### 2.2 Preparation of Cellulose Films Containing Silver Nanoparticles

First, microcrystalline cellulose (particle size: 51 μm, Aldrich) was dried in a vacuum oven at 80 °C under reduced pressure for 24 h. Then, 5.00 g of the dried cellulose were dissolved in 95.00 g of 1-butyl-3-methylimidazolium chloride (BMImCl) at 60 °C under continuous stirring for 24 h, resulting in a 5 wt % cellulose solution in the ionic liquid.

Subsequently, 5.00 g of this solution were poured into a glass Petri dish (9.5 cm in diameter), and a spin coater was used to spread the sample at 500 rpm for 40 s. Afterward, distilled water was added to the Petri dish to regenerate the cellulose as a film. The resulting film was thoroughly washed with water to remove any residual ionic liquid.

Next, 40 mL of each silver nanoparticle dispersion (XmM_AgNP) were added to individual Petri dishes containing wet cellulose films, and the samples were left in contact with the dispersions for 30 days. After this period, the films were rinsed several times with water and air-dried slowly at room temperature for 7 days. The thickness of the films was determined using a digital micrometer by averaging measurements taken at different positions across the samples.

The resulting materials were designated CEL_XmM_AgNP, where X refers to the nominal Ag^+^ concentration in the dispersion. The quantities used in the preparation of the cellulose films are summarized in Table 2.

**Table 2:** Experimental Conditions for the Preparation of Cellulose Films Loaded with AgNPs

Sample ID	AgNP Dispersion Used	Theoretical Amount of Metallic Ag in Dispersion (mg)^a^	Theoretical Ag^0^ Content in Film (%)^b^
CEL_5 mM_AgNP	5 mM_AgNP	21.6 mg	8.00%
CEL_10 mM_AgNP	10 mM_AgNP	43.1 mg	14.7%
CEL_25 mM_AgNP	25 mM_AgNP	107 mg	30.0%
CEL_50 mM_AgNP	50 mM_AgNP	216 mg	46.3%
CEL_100 mM_AgNP	100 mM_AgNP	431 mg	63.3%

a Based on a dispersion volume of 40.0 mL and assuming complete reduction of Ag^+^ to metallic silver (Ag^0^).

b Calculated assuming complete transfer of Ag^0^ from the dispersion to the cellulose film (250 mg).

### 2.3 Catalytic Degradation of 4-Nitrophenol Using Cellulose Films Containing AgNPs

For the catalytic tests, 5.00 mL of an aqueous 3.0 mM 4-nitrophenol (99%, Aldrich) solution was mixed with 5.00 mL of an aqueous 300 mM sodium borohydride (99%, Aldrich) solution in a beaker. A piece of the CEL_XmM_AgNP film with a known mass (approximately 5.00 mg) was then added to the reaction mixture. The concentrations of the reactants were selected based on previous reports in the literature.^30^ The reaction was carried out under magnetic stirring at room temperature. As a control, the same procedure was performed using a pure cellulose film under identical conditions. UV–vis absorption spectra of aliquots from the reaction medium were recorded every 5 min to monitor the progress of the reaction. For each sample, five consecutive catalytic cycles were performed. Between cycles, the film was carefully removed from the reaction medium using tweezers, rinsed with distilled water, and reused in a new test.

### 2.4 Antibacterial Activity Assay

The antibacterial activity of the cellulose–AgNP films was evaluated against *Escherichia coli* (ATCC 8739) and *Staphylococcus aureus* (ATCC 25923). Bacterial suspensions were adjusted to approximately 1 × 10^8^ CFU mL^–1^. Then, 100 μL of each suspension was inoculated onto sterile BHI (brain heart infusion) agar plates (OXOID) and evenly spread using a sterile Drigalski loop to obtain a homogeneous lawn. The membranes were then cut into circular discs (6 mm in diameter) and sterilized by UV exposure under a laminar flow hood for 15 min on each side to ensure asepsis. Each assay was performed in quintuplicate (n = 5) for each sample and bacterial strain. The plates were incubated at 37 °C for 24 h in a BOD incubator. After incubation, the inhibition zone diameters were measured using a caliper. Each value of inhibition zone diameter was expressed as mean ± standard deviation (n = 5). Raw data analysis was performed using Microsoft Excel version 2604. Data were analyzed using two-way analysis of variance (ANOVA) followed by Tukey’s posthoc test for multiple comparisons. Values of *p* < 0.05 were considered significant. Statistical analyses, as well as the graph, were performed using GraphPad Prism 8 software. All experiments were conducted under aseptic conditions, following standard microbiological procedures to ensure the reliability and reproducibility of the results.^31^

### 2.5 Characterization Techniques

The particle size distribution of the AgNP dispersions was determined by dynamic light scattering (DLS), while the zeta potential was measured by laser Doppler electrophoresis, both using Zetasizer Nano (Malvern Instruments, λ = 633 nm). DLS analyses were performed using standard disposable polystyrene cuvettes, whereas zeta potential measurements were carried out in a dedicated folded capillary cell (DTS1070, Malvern). All measurements were conducted in triplicate.

UV–Vis absorption spectra were recorded using an Ocean Optics USB4000 spectrophotometer. For all liquid samples, a 1 cm path length quartz cuvette was used. Spectra were acquired in absorbance mode. To perform the UV–Vis measurements, all AgNP dispersions were first diluted 10 times. Then, depending on the concentration of each sample, additional dilutions were made to keep the absorbance values within the ideal measurement range.

The infrared spectrum of the films was obtained using a Cary 630 FTIR spectrometer (Agilent) equipped with an ATR (Attenuated Total Reflectance) accessory. Spectra were recorded in the range of 650 to 4000 cm^–1^, with a resolution of 4.0 cm^–1^.

X-ray diffraction (XRD) patterns of the film samples were obtained using a MiniFlex benchtop X-ray diffractometer (Rigaku). The instrument operated with Cu–Kα radiation (λ = 1.518 Å) at 30 kV and 15 mA. Scans were performed over a 2θ range of 5.0° to 80.0°, with a step size of 0.02° per second.

Thermogravimetric analyses were performed using an SDT650 thermal analyzer (TA Instruments). The experiments were conducted under a synthetic air atmosphere with a flow rate of 100 mL/min. A platinum crucible was used, and the samples were heated from 30 to 1000 °C at a heating rate of 10 °C/min.

ICP-OES measurements were performed using a Spectro Arcos spectrometer with radial viewing (SOP configuration). The instrument operated with an argon plasma source and was equipped with a solid-state charge-coupled device (CCD) detector. Sample preparation involved digestion with aqua regia, followed by heating in a DigiPREP digestion block (SCP Science).

Transmission electron microscopy (TEM) images were acquired using a JEM-2100F microscope (JEOL). The instrument operated at an accelerating voltage of 200 kV. For sample preparation, a drop of the AgNP dispersion was deposited onto a carbon-coated copper grid and dried under ambient conditions. Particle size analysis and histogram generation were performed using ImageJ software by measuring approximately 200 individual nanoparticles for each sample.

Scanning electron microscopy (SEM) images were acquired using a JSM-7401F microscope (JEOL). The microscope was equipped with an energy-dispersive X-ray spectroscopy (EDS) microprobe (Noran). For analysis, the film samples were sputter-coated with a thin layer of gold to improve conductivity. EDS analysis was performed in both mapping and line scan modes to evaluate the elemental distribution across the samples.

## 3 Results and Discussion

### 3.1 Preparation and Characterization of Highly Concentrated Silver Nanoparticle Dispersions

The silver nanoparticle dispersions were prepared via chemical reduction of silver salt using tetrabutylammonium borohydride (TBABH_4_) in water in the presence of polyvinylpyrrolidone (PVP) as a stabilizing agent. The choice of AgBF_4_ instead of the more commonly used AgNO_3_ was based on literature reports indicating that AgBF_4_ leads to the formation of smaller AgNPs in aqueous PVP systems, likely due to specific interactions between PVP and the tetrafluoroborate anion.^32^ In addition, TBABH_4_ was chosen over NaBH_4_ due to its milder and more selective reducing behavior, particularly during nucleation, which favors the formation of more uniform nanoparticles.^33^ This is a critical factor in our approach, which aims to produce AgNPs at high metal concentrations while maintaining stability and size control. PVP was used due to its known ability to provide steric stabilization, as well as its low toxicity, cost, and commercial availability.^34,35^

Five dispersions were synthesized with initial AgBF_4_ concentrations of 5, 10, 25, 50, and 100 mM, labeled as XmM_AgNP. All samples exhibited a color transition from pale yellow (5 mM) to deep orange (100 mM), as shown in Figure 1, consistent with nanosilver formation. It is worth noting that attempts to prepare samples at concentrations above 100 mM or with PVP content below 10 wt % led to unstable systems with rapid sedimentation or color change.

**Figure 1:**
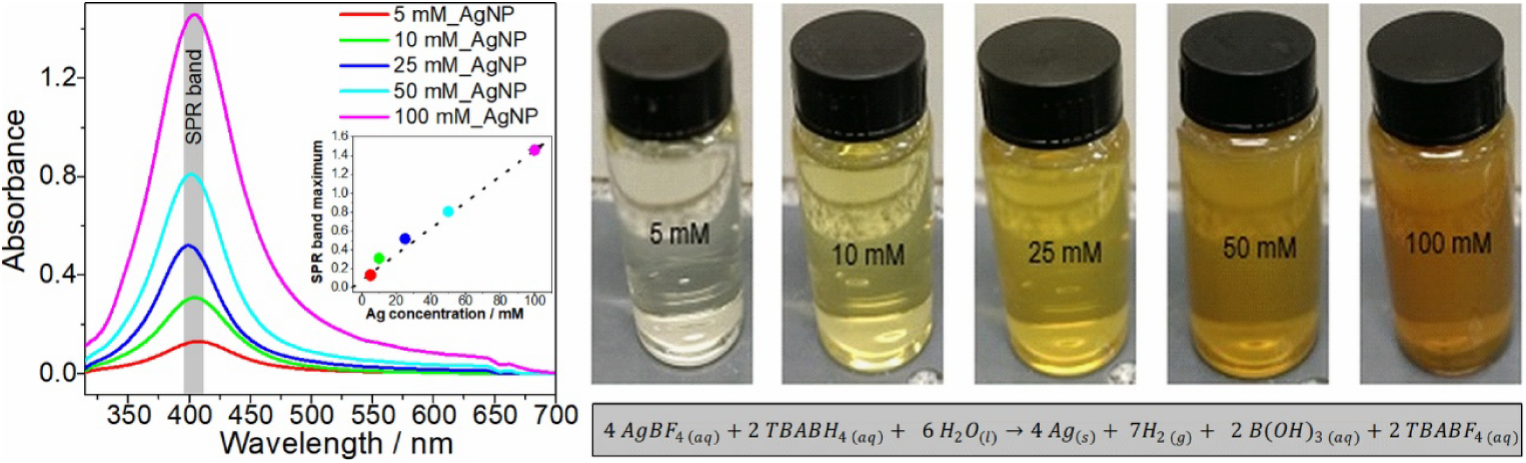
UV–Vis spectra of AgNP dispersions at different precursor concentrations, with the inset showing SPR band intensity vs Ag concentration (left). Representative photographs of the colloidal suspensions are shown on the right; for clarity, the images were taken using diluted samples (300 μL of colloid in 20 mL of distilled water). The chemical equation below describes AgNP formation by reduction of AgBF_4_ with TBABH_4_.

The UV–Vis absorption spectra (Figure 1) showed a surface plasmon resonance (SPR) band centered around 400 nm for all samples, characteristic of metallic silver nanoparticles.^36^ As expected, the SPR intensity increased with increasing AgBF_4_ concentration, while the full width at half-maximum (fwhm) of the normalized spectra remained similar across the series (Figure S1-Supporting Information). This suggests that no marked broadening of the plasmon band occurred across the series, despite the increase in Ag concentration.^37,38^ In addition, these samples were monitored over 1 month at two-day intervals, and the spectra showed only slight variations over time, indicating overall stability of the dispersions (Figure S2-Supporting Information).

Zeta potential measurements were performed to evaluate surface charge and provide complementary information on the colloidal stabilization mechanism of the dispersions (Figure 2A/B). All samples exhibited negative zeta potential values, ranging from −6.7 to −12.8 mV. Although these magnitudes are relatively low and do not suggest strong electrostatic stabilization, they do not necessarily indicate poor colloidal stability. In this case, the stability of the AgNP dispersions is more reasonably attributed to steric stabilization promoted by PVP, a nonionic polymer adsorbed on the nanoparticle surface, which hinders particle–particle approach and reduces aggregation.^39^ This interpretation is consistent with the UV–vis results, which indicate that the dispersions remained stable over time. The negative zeta potential values may arise from the adsorption of anionic species present in the synthesis medium, such as BF_4_^–^, onto the nanoparticle surface.

**Figure 2:**
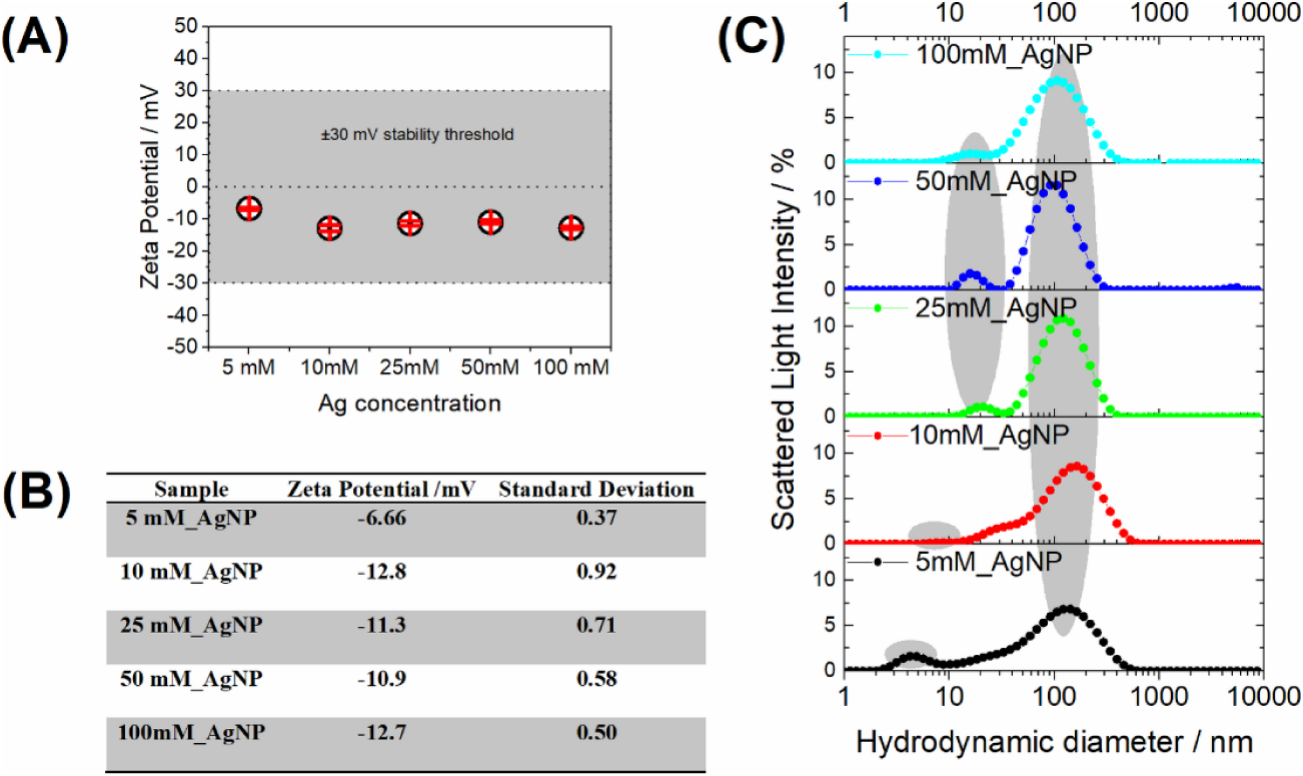
Colloidal stability and hydrodynamic size distribution of AgNP dispersions. (A) Zeta potential values for AgNP samples at different silver concentrations, with the ±30 mV region highlighted as the general threshold for electrostatic colloidal stabilization. (B) Table summarizing the numerical zeta potential values with corresponding standard deviations. (C) Hydrodynamic diameter distributions obtained by dynamic light scattering (DLS), illustrating the size populations of each dispersion.

Dynamic light scattering (DLS) analysis was used to evaluate the hydrodynamic size distributions of the AgNP dispersions (Figure 2C). The intensity-weighted profiles exhibited bimodal distributions for all samples, indicating the presence of at least two hydrodynamic populations. Because DLS provides hydrodynamic diameters and the scattered intensity scales with the sixth power of particle radius (R^6^), the larger-size population can be disproportionately influenced by a relatively small fraction of larger species, such as PVP-coated assemblies or small aggregates.^40^ Therefore, these distributions were interpreted qualitatively as evidence of multimodal colloidal populations rather than as a direct measure of nanoparticle core-size homogeneity. In general, the lower-diameter population was shifted to smaller values for the 5 and 10 mM samples, whereas broader distributions became more evident as the precursor concentration increased, particularly for the 100 mM sample.

Transmission electron microscopy (TEM) images (Figure 3) provided a more direct assessment of nanoparticle morphology and core size. Representative size histograms were obtained by measuring approximately 200 nanoparticles for each sample. For the 5 and 10 mM samples (Figure 3A/B), predominantly spherical nanoparticles with diameters mainly in the 5–8 nm range were observed. For the 25 and 50 mM samples (Figure 3C/D), the particles remained mostly spherical, with a shift toward slightly larger diameters, typically around 8–12 nm. At 100 mM (Figure 3E), a broader size distribution was observed, indicating reduced size uniformity at the highest precursor concentration. Some irregular particles and small aggregates are visible in the micrographs; however, only clearly distinguishable individual nanoparticles were considered for histogram construction. High-resolution TEM (Figure 3F) confirmed the crystalline nature of the AgNPs. Lattice fringes with 0.24 nm spacing were observed, corresponding to the (111) plane of the face-centered cubic (FCC) silver structure. This is consistent with the expected crystal structure of metallic silver synthesized via chemical reduction (JCPDS: 4-0783).

**Figure 3:**
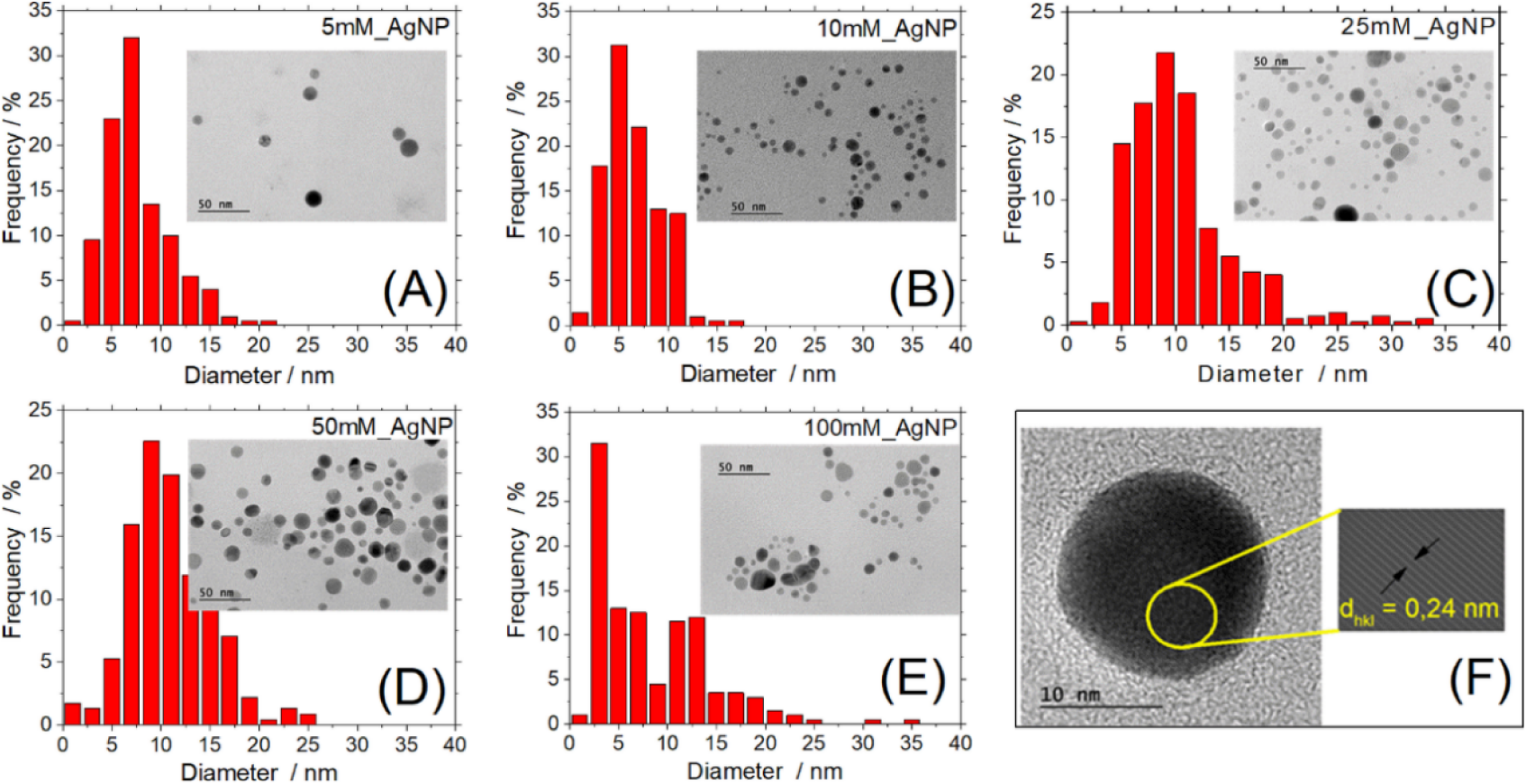
Particle size distribution histograms and TEM images of silver nanoparticles (AgNPs) synthesized at different precursor concentrations. (A–E) Histograms and representative TEM micrographs of AgNPs synthesized with Ag^+^ precursor concentrations of 5, 10, 25, 50, and 100 mM, respectively. Histograms were constructed by measuring approximately 200 individual nanoparticles for each sample using ImageJ. (F) High-resolution TEM image of an individual particle showing lattice fringes with interplanar spacing (d111 = 0.24 nm) corresponding to the (111) plane of face-centered cubic silver.

Overall, the TEM analysis complements the DLS results by showing that, despite the multimodal hydrodynamic distributions observed by DLS, the samples are predominantly composed of small, mostly spherical AgNPs, with broader size distributions becoming more evident at higher precursor concentrations. This apparent difference is expected because DLS measures hydrodynamic size in dispersion and is highly sensitive to a minor fraction of larger species, whereas TEM directly visualizes the metallic cores.

In summary, the PVP-stabilized route enabled the synthesis of AgNP dispersions with concentrations up to 100 mM, while maintaining colloidal stability for at least 1 month. The nanoparticles were predominantly small, spherical, and crystalline, as supported by UV–Vis, zeta potential, DLS, and TEM analyses. Although broader size distributions became more evident at higher precursor concentrations, particularly at 100 mM, the results indicate that this strategy provides concentrated AgNP dispersions with preserved colloidal and structural characteristics suitable for subsequent incorporation into cellulose films.

### 3.2 Preparation and Characterization of the Cellulose Film Containing Silver Nanoparticles

The AgNP dispersions prepared in the first part of this work were subsequently used for *ex situ* impregnation of regenerated cellulose films, enabling evaluation of the resulting materials as (i) heterogeneous catalysts and (ii) antimicrobial films. The availability of concentrated and colloidally stable AgNP dispersions was advantageous because it enabled nanoparticle incorporation into the cellulose matrices without additional concentration or solvent-removal steps prior to impregnation.

Cellulose-based films were prepared through a three-step process involving cellulose dissolution in BMImCl, film formation by regeneration, and subsequent nanoparticle impregnation (Figure 4A). This procedure, which is well established in our research group,^41–43^ yielded water-insoluble regenerated cellulose films suitable for AgNP uptake. The incorporation process was monitored by UV–Vis absorption spectroscopy, using the 5 mM_AgNP dispersion as a model system. A gradual decrease in the characteristic SPR band at 400 nm over 15 days confirmed the progressive uptake of nanoparticles by the film, reaching a plateau indicative of saturation (Figure 4B). To ensure complete incorporation, all films were kept in contact with their respective dispersions for 30 days. After impregnation, the films exhibited a characteristic yellowish color (Figure 4D) consistent with the presence of AgNPs. The resulting materials were named CEL_xmM_AgNP, where x corresponds to the concentration of the initial AgNP dispersion. These samples were systematically characterized by FTIR, XRD, TGA, and ICP-OES. Film thickness was measured at ten different points using a digital micrometer, yielding an average value of 0.010 ± 0.001 mm for all samples. In addition, UV–Vis spectroscopy was performed for CEL_5 mM_AgNP, the most transparent film, which displayed a distinct SPR band at 410 nm, confirming successful nanoparticle incorporation (Figure 4C). In contrast, pure cellulose films showed no absorption in the visible range, and higher-loading films were too opaque for UV–Vis analysis.

**Figure 4:**
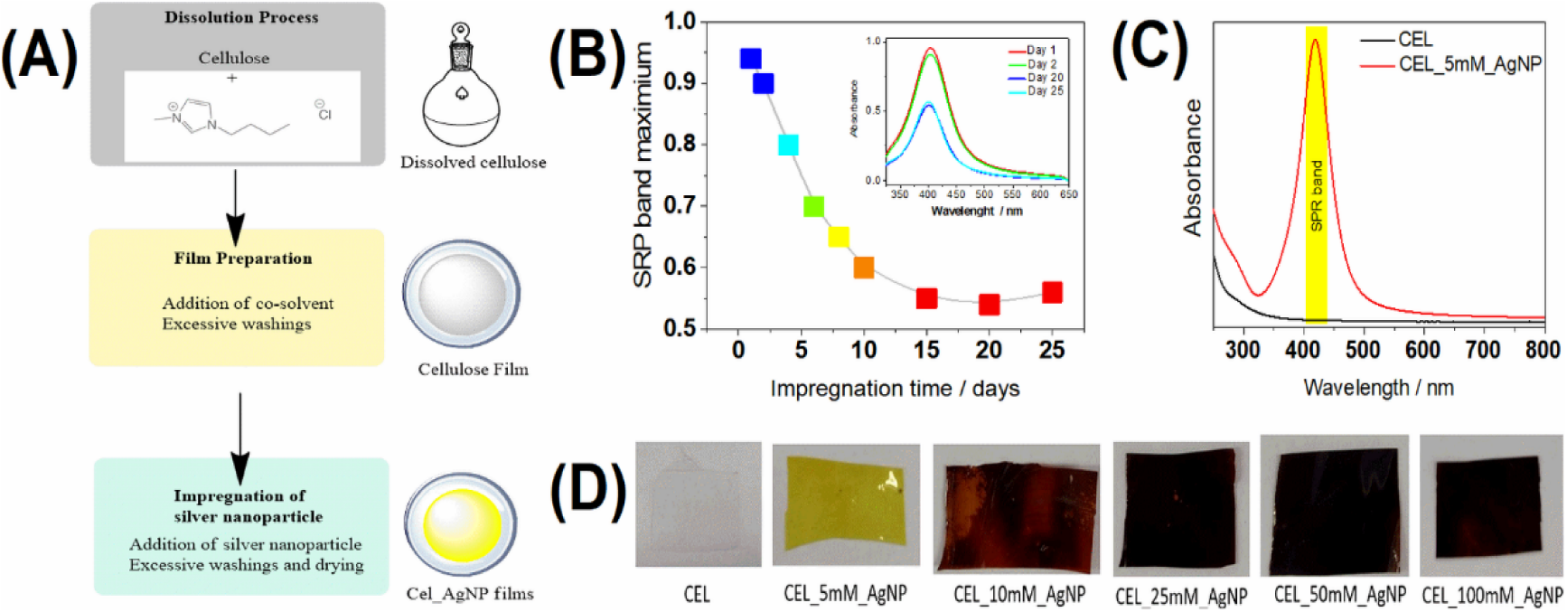
(A) Schematic process for the preparation of AgNP-impregnated cellulose films. (B) Monitoring of silver nanoparticle impregnation in cellulose films by UV–Vis spectroscopy over 30 days. (C) Comparison of the UV–Vis spectra of a cellulose film containing AgNPs (CEL_5 mM_AgNP) with pure cellulose and (D) photographs of CEL_xmM_AgNP samples.

The FTIR spectra of all films are shown in Figure 5, divided into two regions for clarity: 800–2000 cm^–1^ (Figure 5A) and 2000–4000 cm^–1^ (Figure 5B). The spectrum of the pure cellulose film (black) displays characteristic bands of regenerated cellulose,^44,45^ including broad O–H stretching (∼3350 cm^–1^), O–H bending (∼1650 cm^–1^), C–H stretching (∼2950 cm^–1^), and intense C–O stretching in the 1000–1200 cm^–1^ region. The spectra of AgNP-impregnated films (colored traces) retain all major cellulose bands, confirming structural preservation. Additional bands at 2970 and 1430 cm^–1^ (C–H stretching and bending), 1660 cm^–1^ (amide C=O stretching), and 1290 cm^–1^ (C–N stretching) are attributed to PVP,^46^ indicating its coimpregnation with AgNPs. These PVP bands were also present in the film exposed to PVP solution without AgNPs (red trace), confirming PVP incorporation during the impregnation process.

**Figure 5:**
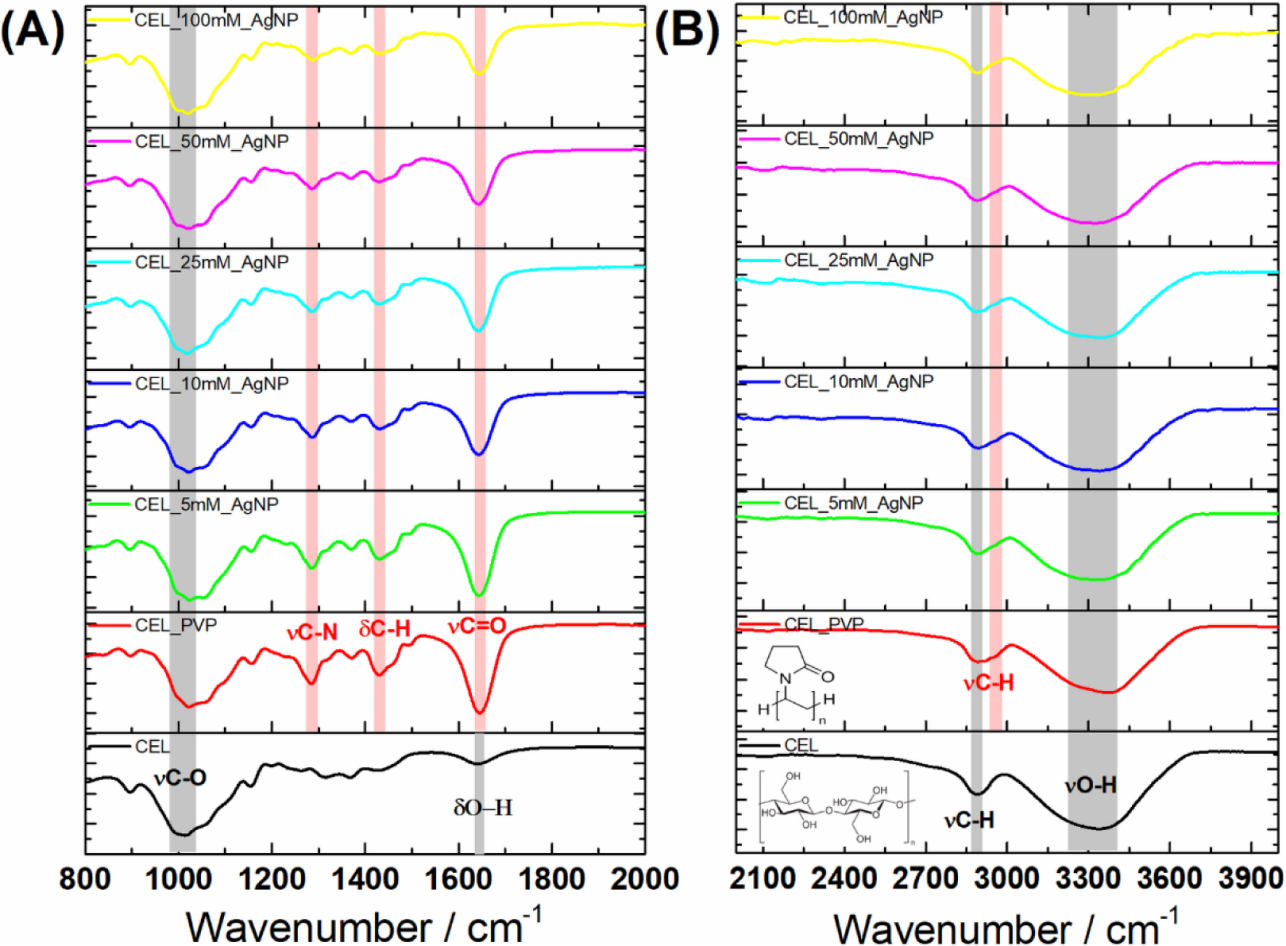
FTIR spectra of pure cellulose (CEL), CEL_PVP, and AgNP-impregnated cellulose films at different precursor concentrations. (A) Region (800–2000 cm^–1^) and (B) region (2100–4000 cm^–1^), highlighting characteristic functional group vibrations.

Figure 6 shows the XRD patterns of the pure cellulose film (CEL), the PVP-treated film (CEL_PVP), and the AgNP-loaded films (CEL_xmM_AgNP). The diffraction pattern of the pure cellulose film (black trace) exhibits a broad, low-intensity halo centered at 2θ ≈ 21°, associated with the (020) reflection of cellulose type II.^47^ This low crystallinity is attributed to the disruption of the crystalline regions of microcrystalline cellulose during dissolution and regeneration in ionic liquid.^44,48^ All AgNP-impregnated films (colored traces) retain this broad signal, indicating that the cellulose remains in the type II form. In addition, diffraction peaks at 2θ ≈ 38°, 44°, 64°, and 77° are observed, corresponding to the (111), (200), (220), and (311) planes of face-centered cubic (fcc) metallic silver (JCPDS: 4-0783). The intensity of the Ag(111) reflection at 2θ ≈ 38° varied among the films, and in some cases, the peak profile also appeared broader. These differences may be associated with variations in the effective amount of silver incorporated into the cellulose/PVP matrix, as well as with differences in the distribution, crystallite size, and structural order of the Ag-containing domains. Therefore, the Ag(111) peak was interpreted qualitatively as evidence of metallic silver incorporation rather than as a strictly proportional measure of Ag loading. No additional peaks are observed in the CEL_PVP sample, consistent with the amorphous nature of PVP (Figure S3-Supporting Information).

**Figure 6:**
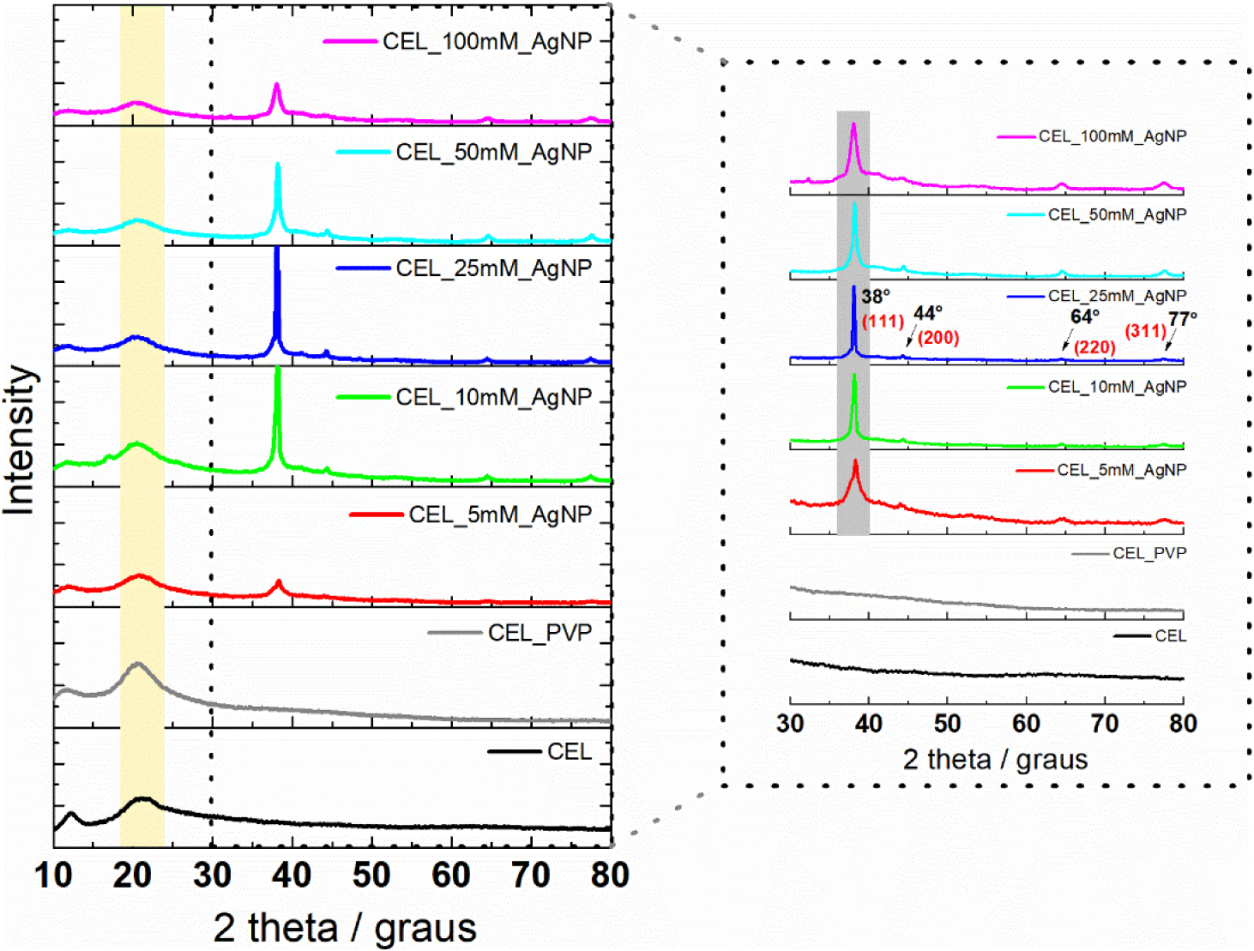
X-ray diffraction patterns of pure cellulose (CEL), cellulose/PVP (CEL_PVP), and cellulose films containing different concentrations of silver nanoparticles (CEL_xmM_AgNP). On the right, the enlarged region between 30 and 80° (2θ) is shown, highlighting the characteristic peaks of metallic silver corresponding to the (111), (200), (220), and (311) planes.

Figure 7A shows the thermogravimetric (TG) and Figure 7B the derivative (DTG) curves of the pure cellulose film (CEL), the film exposed to PVP solution (CEL_PVP), and the AgNP-impregnated films (CEL_xmM_AgNP). The CEL film exhibited three main mass loss events: an initial ∼5% loss between 25 and 150 °C due to water desorption; a peak at 290 °C associated with degradation of amorphous cellulose regions; and a second peak near 520 °C related to crystalline cellulose degradation.^49^ AgNP-impregnated films showed increased water loss in the 25–100 °C range compared to cellulose film, attributed to the presence of PVP, a more hygroscopic polymer. The onset of thermal degradation was shifted to higher temperatures (∼320–350 °C) compared with the pure cellulose film, indicating enhanced thermal stability due to PVP incorporation.^50^ This result agrees with that observed for the CEL_PVP film (gray curve), supporting that interactions between cellulose and PVP are responsible for the higher thermal stability, as illustrated in Figure S4-Supporting Information. Additionally, all AgNP-containing films exhibited an extra degradation event between 390 and 450 °C (highlighted in green), absent in pure cellulose and consistent with PVP decomposition, typically observed near 420 °C.^51^ At higher temperatures (450–580 °C), a broader degradation region was observed, likely corresponding to overlapping degradation of both cellulose and PVP. At 800 °C, the AgNP films presented residual mass ranging from 4% to 8%, attributed to metallic silver (Table S1-Supporting Information). These values were lower than the nominal silver contents estimated for each sample, suggesting a limited capacity for AgNP incorporation into the cellulose matrix.

**Figure 7:**
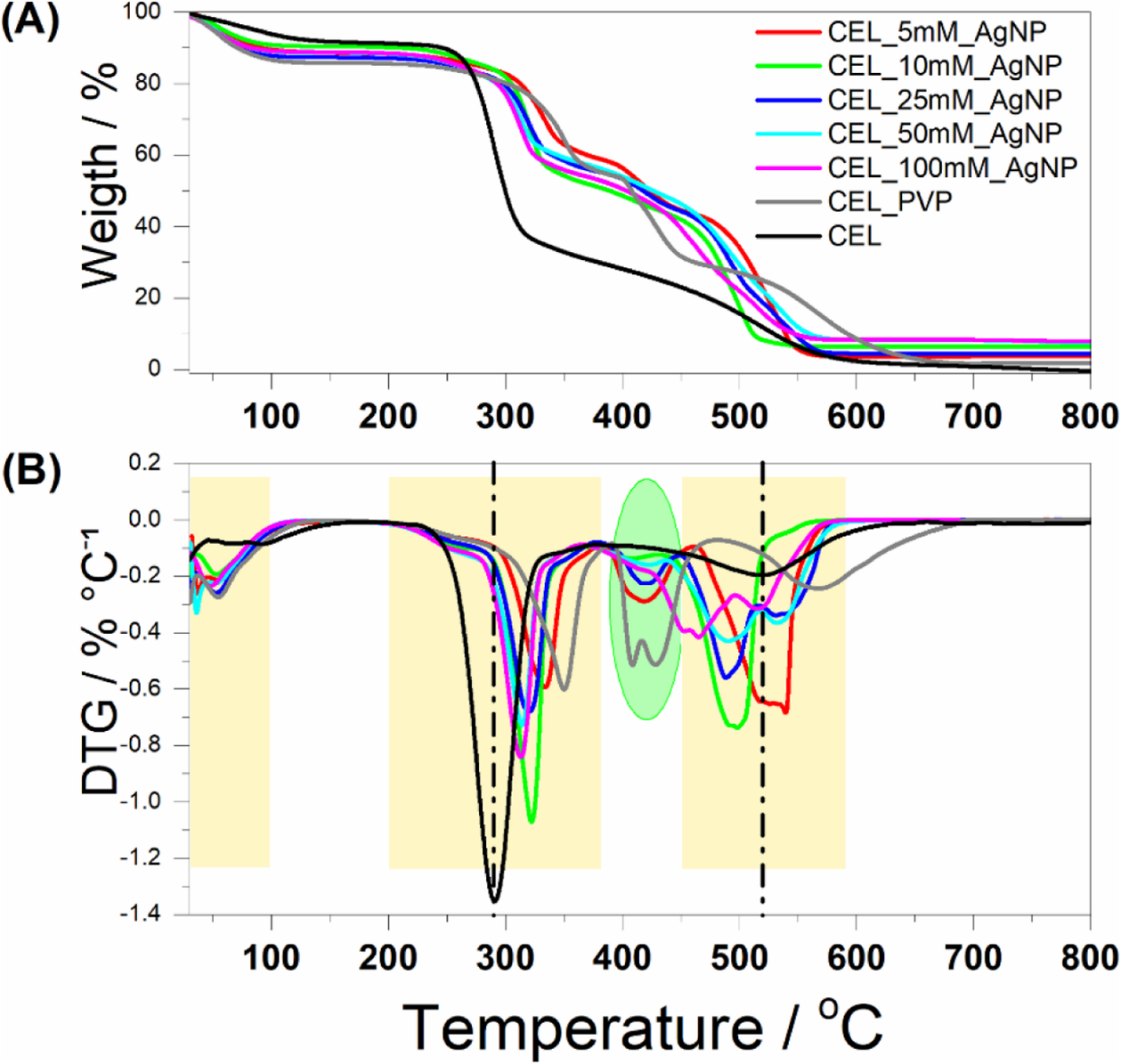
(A) TGA and (B) DTG curves of pure cellulose and AgNP-impregnated cellulose films at different precursor concentrations, showing thermal stability and decomposition profiles. The highlighted yellow regions correspond to the main stages of cellulose thermal decomposition, while the green region represents the decomposition of PVP.

Consistent with this interpretation, ICP-OES analysis of the CEL_xmM_AgNP films revealed a gradual increase in silver content with increasing concentration of the impregnation dispersions, reaching 1.47% for CEL_5 mM_AgNP, 2.53% for CEL_10 mM_AgNP, 3.06% for CEL_25 mM_AgNP, 3.10% for CEL_50 mM_AgNP, and 4.17% for CEL_100 mM_AgNP. Although the values increase with concentration, they remain below the estimated nominal values (Table 2), reinforcing the interpretation that silver incorporation into the cellulose matrix tends toward a saturation behavior.

The silver content in the CEL_xmM_AgNP films increased with the concentration of the impregnation dispersion, although this increase was not proportional. In particular, the small difference between the 25 and 50 mM samples, together with the only moderate increase observed for the 100 mM sample, suggests that silver uptake becomes progressively limited as the concentration of the dispersion increases. This behavior indicates that Ag incorporation depends not only on the concentration of the starting colloid but also on the limited capacity of the regenerated cellulose/PVP matrix to accommodate and retain Ag-containing domains. Because the nanoparticles are stabilized by PVP, incorporation is expected to depend on the interaction between the PVP-coated nanoparticles and the cellulose matrix. In this context, steric effects associated with the polymer shell are likely to reduce incorporation efficiency, particularly at higher external AgNP concentrations. This interpretation is further supported by an additional experiment using a much higher-molar-mass PVP stabilizer (Mw ≈ 360000), in which Ag incorporation into the films was markedly hindered. Overall, these results indicate that the final silver loading is controlled by matrix-limited retention and steric constraints, rather than solely by the nominal concentration of the impregnation dispersion.

SEM micrographs of the CEL_PVP, CEL_5 mM_AgNP, and CEL_100 mM_AgNP films are shown in Figure 8. Additional micrographs, including those of films prepared with intermediate AgNP concentrations, are provided in Figure S5-Supporting Information. The CEL_PVP sample exhibited a continuous but textured surface morphology, which is consistent with regenerated cellulose films obtained from dissolution and coagulation processes in ionic-liquid-based systems.^52,53^ The CEL_PVP sample did not show the bright contrast features observed in the Ag-impregnated films. In contrast, the Ag-containing films displayed bright surface features, which became more abundant in the film prepared from the more concentrated AgNP dispersion. At higher magnification, the CEL_100 mM_AgNP film exhibited numerous rounded bright surface features, including a large population of smaller domains accompanied by less frequent larger ones. These contrast features are consistent with silver-containing surface domains formed after impregnation. Their coexistence suggests a heterogeneous surface population, including finer Ag-rich features together with larger Ag-enriched domains.

**Figure 8:**
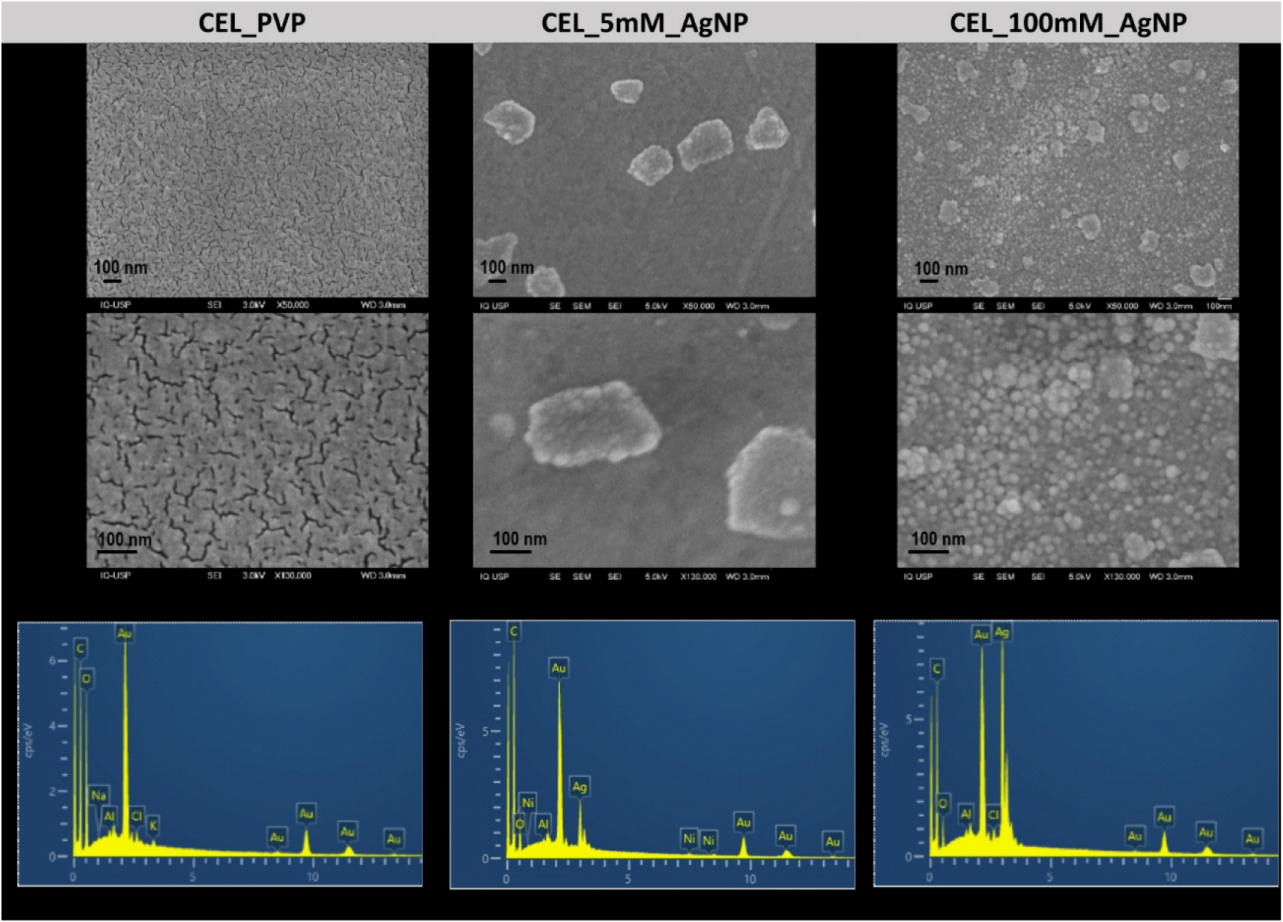
SEM micrographs at 5000× and 13000× magnification and corresponding EDS spectra of CEL_PVP, CEL_5 mM_AgNP, and CEL_100 mM_AgNP films. The reference CEL_PVP film exhibits continuous surface morphology, whereas the AgNP-containing films show bright surface contrast features and detectable Ag signals in EDS. The Ag signal is more evident in CEL_100 mM_AgNP. Au peaks arise from the conductive gold coating used for SEM analysis.

EDS analyses supported this interpretation. The CEL_PVP film showed only the expected signals of the cellulose/PVP matrix, whereas the Ag-containing films exhibited detectable Ag signals, confirming the presence of silver species after impregnation. The Ag signal was more evident for CEL_100 mM_AgNP than for CEL_5 mM_AgNP, in agreement with the higher silver incorporation determined by ICP-OES. Au signals in the spectra arise from the conductive gold coating used for SEM sample preparation. Overall, the SEM/EDS results provide complementary evidence for the successful incorporation of silver-containing domains into the cellulose/PVP films.

Additional support for Ag incorporation was obtained from EDS elemental mapping (Figure S6-Supporting Information). While the reference CEL_PVP film showed only C and O distributions, the Ag-impregnated films exhibited detectable Ag throughout the analyzed areas. Moreover, the Ag signal was more intense for CEL_100 mM_AgNP than for CEL_5 mM_AgNP, consistent with the ICP-OES results. This interpretation is further supported by EDS line-scan analyses (Figure S7-Supporting Information). Detectable Ag signals were observed along the analyzed paths for both CEL_5 mM_AgNP and CEL_100 mM_AgNP, with local intensity fluctuations along the scan direction.

Overall, the results demonstrate that regenerated cellulose films prepared from nonderivatized cellulose in ionic liquid can act as effective supports for the ex-situ immobilization of PVP-stabilized AgNPs. Although the Ag content increased with the concentration of the impregnation dispersion, the uptake was not proportional, indicating a saturation-limited process governed by the finite capacity of the cellulose matrix to retain Ag-containing domains. The proposed strategy combines colloidal AgNP systems with a renewable, water-insoluble, and easily recoverable cellulose film platform. These features are particularly relevant for the development of multifunctional materials intended for catalytic and antibacterial applications, where immobilization, stability, and reusability are key requirements.

### 3.3 Catalytic Activity

The catalytic activity of the AgNP-loaded cellulose films was evaluated through the model reduction of 4-nitrophenol (4-NP) to 4-aminophenol (4-AP) in the presence of excess NaBH_4_. This reaction is both industrially relevant and environmentally significant, as it converts a toxic nitro compound into a valuable pharmaceutical intermediate.^54,55^ The progress was monitored by UV–Vis spectroscopy, which allows real-time tracking through the disappearance of the absorption band at 396 nm (4-nitrophenolate ion) and the emergence of a new band at 298 nm, characteristic of 4-AP. In a typical experiment, aqueous solutions of 4-NP (3.00 mM) and NaBH_4_ (300 mM) were mixed, and approximately 5.00 mg of film was added. The large excess of reducing agent ensured pseudo-first-order kinetics. After each cycle, the film was easily removed with tweezers, rinsed with water, and reused. Recyclability was evaluated over five consecutive cycles, in line with recent studies on supported AgNP catalysts for the reduction of 4-nitrophenol.^56–60^ A control experiment using a pure cellulose film (without AgNPs) showed no catalytic activity, as confirmed by the unchanged UV–Vis spectra even after 24 h of reaction (data not shown).

To illustrate the catalytic performance of the AgNP-containing films, Figure 9A presents the UV–Vis spectra for the first catalytic cycle using the CEL_5 mM_AgNP film. A gradual decrease in the absorption band at 396 nm and the appearance of a new band at 298 nm confirms the efficient conversion of 4-NP into 4-AP. The conversion profiles over five consecutive cycles for this film are shown in Figure 9B. Although the first cycle showed the highest conversion, the subsequent cycles did not follow a strictly monotonic trend and instead fluctuated within a relatively narrow range while maintaining high overall conversion. Therefore, rather than indicating progressive improvement or deterioration of the film surface, these data suggest an initial decrease in activity followed by stabilization with small cycle-to-cycle variations.

**Figure 9:**
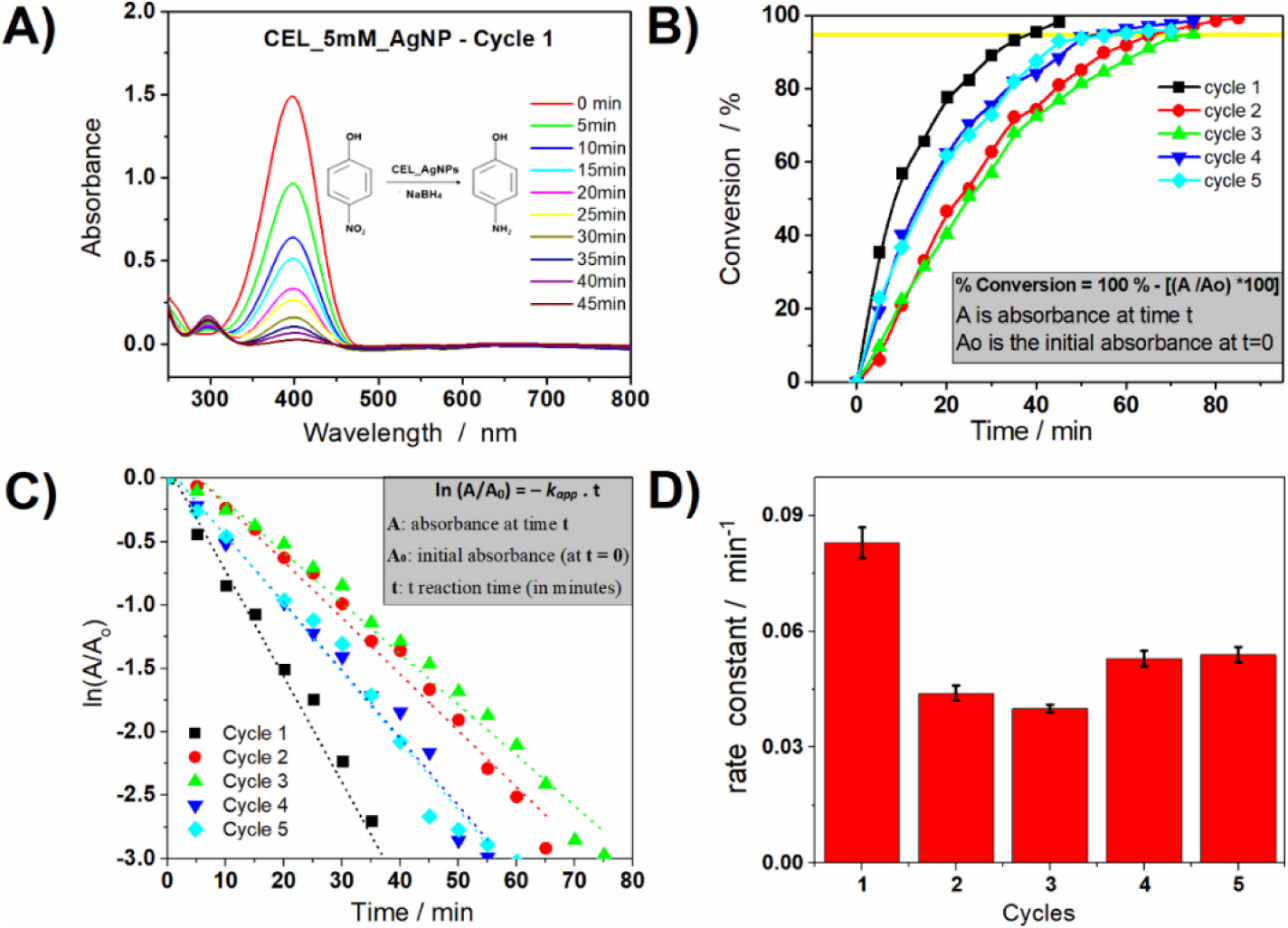
Catalytic performance of CEL_5 mM_AgNP films in the reduction of 4-nitrophenol over five consecutive cycles. (A) UV–Vis absorption spectra during the first cycle, showing the progressive decrease of the 400 nm band. (B) Conversion profiles as a function of time for cycles 1–5. (C) Pseudo-first-order kinetic plots of ln­(A/A_0_) versus time for cycles 1–5, with linear fits showing correlation coefficients (R^2^) of 0.977, 0.980, 0.981, 0.981, and 0.981, respectively. (D) Apparent rate constants (k_app) for each cycle. Error bars correspond to the standard errors of the fitted slopes obtained from linear regression.

The ln­(A/A_0_) versus time plots for CEL_5 mM_AgNP showed linear profiles in all catalytic cycles, with high correlation coefficients (R^2^ ≥ 0.98), confirming pseudo-first-order kinetics under the evaluated conditions. The apparent rate constants (k_app), obtained from the slopes of these fits, showed a marked decrease from the first to the second cycle, followed by a more stable regime with modest fluctuations over the subsequent cycles. Since each condition was evaluated in a single kinetic run, the uncertainties correspond to the standard errors of the fitted slopes. The higher activity in the first cycle may be related to an initially cleaner and more accessible AgNP surface, whereas the later fluctuations likely reflect minor differences in film handling and contact with the reaction medium rather than a continuous loss of intrinsic catalytic activity. Similar kinetic and reusability profiles were observed for the other AgNP-containing films (Figure S8-Supporting Information).

Figure 10 shows the effect of AgNP loading on the catalytic performance of the films over five consecutive cycles. In the first cycle, films prepared with higher AgNP concentrations (25–100 mM) showed higher apparent rate constants (*k*_app_ > 0.15 min^–1^), indicating the beneficial effect of increased silver loading on catalytic performance. Even at lower loadings (5 and 10 mM), the films exhibited measurable catalytic activity. To allow a more appropriate comparison among the formulations, the first-cycle k_app values were also normalized to the actual silver mass present in each film sample, as determined by ICP-OES. The resulting normalized values were 18.82, 16.86, 16.99, 18.71, and 11.35 s^–1^ g^–1^ for the 5, 10, 25, 50, and 100 mM films, respectively. Although higher Ag loading generally increased the absolute reaction rate, the Ag-normalized catalytic efficiencies remained within a relatively narrow range for the 5–50 mM films and were lower only for the 100 mM sample, suggesting a tendency toward saturation at higher loadings. This behavior suggests that the additional incorporated silver did not translate proportionally into accessible catalytic sites, possibly due to partial inaccessibility of Ag species and/or mass-transfer limitations at the highest loading. After the first cycle, a decrease in activity was observed for all samples, followed by a more stable regime with modest fluctuations over the subsequent cycles. Overall, the films retained measurable catalytic activity over five consecutive cycles, indicating short-term reusability.

**Figure 10:**
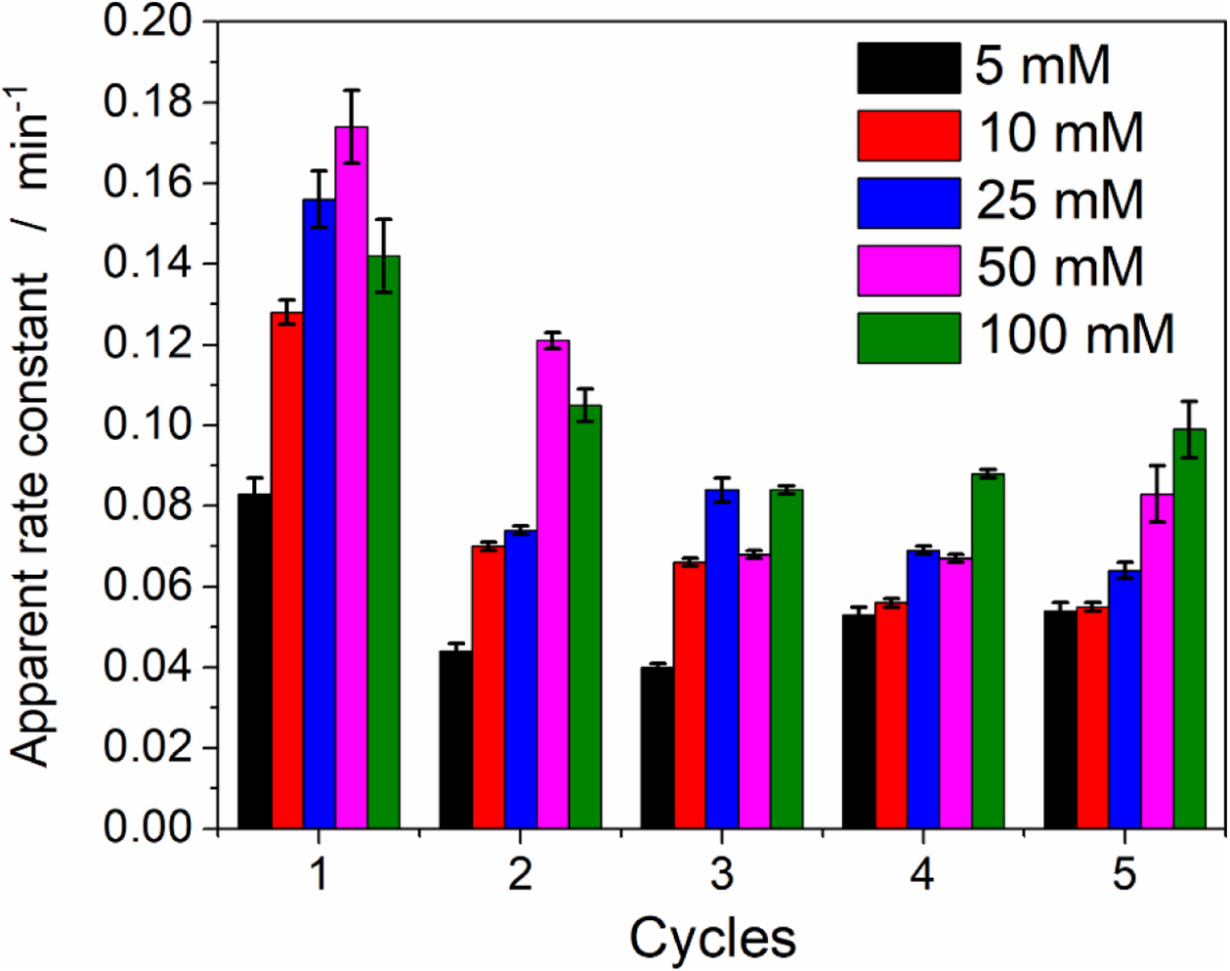
Apparent rate constants (k_app) for the reduction of 4-nitrophenol catalyzed by CEL_xmM_AgNP films over five consecutive cycles. Error bars correspond to the standard errors of the fitted slopes obtained from linear regression.

To further assess catalyst stability, ICP-OES analysis of the reaction supernatant was carried out after the first and fifth catalytic cycles using the CEL_50 mM_AgNP film, selected because of its high catalytic performance. Silver losses corresponding to 9.5% and 1.0% of the initial Ag content of the film were detected in the supernatants collected after the first and fifth cycles, respectively. The higher Ag release during the first run is consistent with the marked decrease in catalytic activity observed after the initial cycle and is likely associated with the removal of weakly bound or more surface-accessible silver species. In contrast, the much lower Ag release after the fifth cycle (<0.2 ppm in solution) indicates that the remaining AgNPs were more effectively retained within the cellulose/PVP matrix. This behavior supports an initial stabilization or conditioning of the catalyst surface, followed by improved operational stability during reuse. The maintenance of complete catalytic conversion over five consecutive cycles further reinforces the suitability of the AgNP-loaded cellulose film for repeated catalytic use.

### 3.4 Antibacterial Activity

The antibacterial performance of the CEL_xmM_AgNP films was assessed against *Escherichia coli* and *Staphylococcus aureus* using the standard disk diffusion method (Figure 11). For the assays, the films were cut into circular disks with a diameter of 6.0 mm and placed on agar plates inoculated with the bacterial strains. A CEL_PVP disk (6.0 mm) was used as a negative control. Therefore, only inhibition halos with diameters larger than 6.0 mm were considered evidence of antibacterial activity. Inhibition zone diameters are reported as mean ± standard deviation (n = 5). Statistical analysis was performed by two-way ANOVA followed by Tukey’s post hoc test for multiple comparisons, and differences were considered significant when *p* < 0.05.

**Figure 11:**
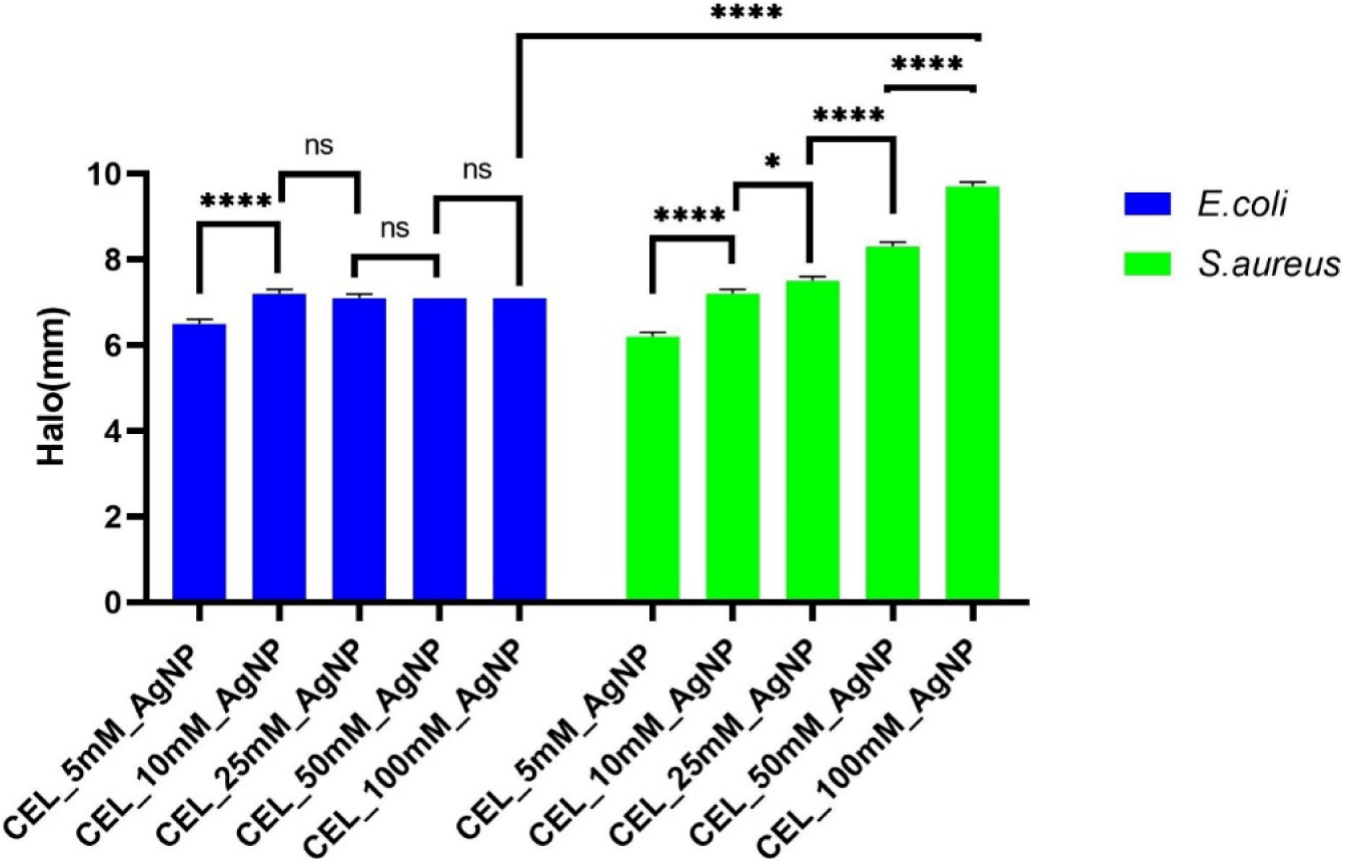
Antibacterial activity of CEL_xmM_AgNP films against *Escherichia coli* and *Staphylococcus aureus* evaluated by the disk diffusion method. Inhibition zone diameters are expressed as mean ± standard deviation (*n* = 5), with error bars representing the standard deviation. CEL_PVP was used as the control, and all the film disks had a diameter of 6.0 mm. Statistical analysis was performed by two-way ANOVA, considering bacterial strain and film formulation as factors, followed by Tukey’s post hoc test for multiple comparisons. Statistical significance is indicated as *p* < 0.05 (*), *p* < 0.01 (**), *p* < 0.001 (***), and *p* < 0.0001 (****); ns indicates no significant difference.

The results revealed a distinct difference in susceptibility between the two strains. For *E. coli*, inhibition halos remained nearly constant across the tested AgNP concentrations, with mean diameters of 6.5 ± 0.1, 7.2 ± 0.1, 7.1 ± 0.1, 7.1 ± 0.0, and 7.1 ± 0.0 mm for CEL_5 mM_AgNP, CEL_10 mM_AgNP, CEL_25 mM_AgNP, CEL_50 mM_AgNP, and CEL_100 mM_AgNP, respectively. Statistical analysis indicated a significant increase from CEL_5 mM_AgNP to CEL_10 mM_AgNP, whereas no significant differences were observed among CEL_10 mM_AgNP, CEL_25 mM_AgNP, CEL_50 mM_AgNP, and CEL_100 mM_AgNP. This behavior suggests that a saturation effect may be reached at relatively low silver contents. It may be attributed to the structural features of Gram-negative bacteria, particularly the outer membrane enriched in lipopolysaccharides, which acts as a selective permeability barrier, as well as to intrinsic defense mechanisms that limit the effectiveness of silver ions.^31,61^

In contrast, *S. aureus* exhibited a concentration-dependent response, with inhibition zone diameters increasing from 6.2 ± 0.1 mm for CEL_5 mM_AgNP to 7.2 ± 0.1, 7.5 ± 0.1, 8.3 ± 0.1, and 9.7 ± 0.1 mm for CEL_10 mM_AgNP, CEL_25 mM_AgNP, CEL_50 mM_AgNP, and CEL_100 mM_AgNP, respectively. Statistical analysis confirmed that the increase in inhibition zone diameter was significant with increasing AgNP concentration, especially from CEL_10 mM_AgNP onward. These results indicate that, under the present experimental conditions, *S. aureus* was more responsive to the AgNP-loaded cellulose films than *E. coli*. This behavior is consistent with the absence of an outer membrane in Gram-positive bacteria and the greater accessibility of the peptidoglycan layer to silver species. It should be noted that the inhibition zones may not result exclusively from direct contact with immobilized metallic AgNPs. Although the ICP-OES leaching experiment was performed in an aqueous catalytic medium and quantifies total Ag rather than Ag^+^ speciation, it indicated that a small fraction of weakly retained silver-containing species can be released from the films. Therefore, bioavailable silver species, potentially including ionic silver species, may also contribute to the antibacterial response observed in the agar disk diffusion assay. Overall, these findings indicate that AgNP-loaded cellulose films exhibited antibacterial activity against both Gram-negative and Gram-positive strains, with a stronger concentration-dependent effect observed for *S. aureus*.

Several recent AgNP–cellulose systems reported in the literature rely on cellulose functionalization, such as dialdehyde/carboxylated or cationized cellulose, to improve AgNP nucleation, anchoring, dispersion, and antimicrobial performance.^62–65^ These studies demonstrate the potential of modified cellulose matrices for preparing antibacterial silver-based materials. Although effective, such approaches generally require additional reagents and extra synthetic or functionalization steps, including prior chemical modification of the cellulose substrate. In contrast, the present work employs highly concentrated and stable preformed AgNP dispersions to impregnate regenerated cellulose/PVP films without prior cellulose derivatization or in situ Ag^+^ reduction. Beyond the simplified preparation route, the obtained films combine reusable catalytic activity toward 4-nitrophenol reduction with antibacterial functionality, highlighting a straightforward multifunctional platform based on renewable cellulose materials.

## 4 Conclusions

This study presents a straightforward strategy for producing highly concentrated and colloidally stable silver nanoparticle dispersions and their subsequent immobilization into regenerated cellulose films prepared from nonderivatized cellulose. The AgNPs were effectively incorporated into the cellulose matrix, reaching silver contents of up to 5 wt %, while preserving the characteristic low-crystallinity cellulose II structure of the regenerated films. Spectroscopic, thermal, microscopic, and elemental analyses confirmed the presence of metallic silver and PVP within the films, as well as enhanced thermal stability compared with pure cellulose. Although Ag incorporation increased with the concentration of the impregnation dispersion, the uptake was not proportional, indicating a saturation-limited process governed by the finite capacity of the cellulose/PVP matrix to retain Ag-containing domains. The resulting AgNP-loaded cellulose films showed significant catalytic activity in the reduction of 4-nitrophenol and retained measurable activity over five consecutive cycles, despite an initial decrease associated with catalyst surface conditioning and partial Ag release. Antibacterial assays also showed activity against both *Escherichia coli* and *Staphylococcus aureus*, with a statistically supported, concentration-dependent response that was more pronounced for the Gram-positive strain. Overall, the combination of concentrated AgNP dispersions with regenerated cellulose films provides recyclable and multifunctional materials that integrate catalytic and antimicrobial properties, contributing to the development of sustainable silver-based nanocomposites.

## Supplementary Material



## Notes

Normalized absorption spectra of silver nanoparticle dispersions (Figure S1); UV–Vis temporal evolution of AgNP dispersions over 30 days for stability evaluation (Figure S2); XRD pattern of pure PVP powder (Figure S3); schematic representation of the interaction between cellulose and PVP (Figure S4); SEM images of PVP and Ag–cellulose films at different magnifications (Figure S5); EDS elemental mapping analyses of CEL_PVP, CEL_5 mM_AgNP, and CEL_100 mM_AgNP films (Figure S6); EDS line-scan analyses of CEL_5 mM_AgNP and CEL_100 mM_AgNP films (Figure S7); kinetic profiles for the catalytic reduction of 4-nitrophenol using Ag–cellulose films (Figure S8); thermogravimetric data of cellulose-based films (Table S1) (PDF)
