# Supplementary material for: Synthesis of Highly Concentrated Silver Nanoparticle Dispersions and Their Integration into Cellulose Films for Catalytic and Antibacterial Applications
Source: ACS Omega. 2026 Jul 22;11(30):44667–81. doi: 10.1021/acsomega.5c13300 (PMC13448974; doi:10.1021/acsomega.5c13300)
Supplement: Supplementary file 1 [file ao5c13300_si_001.pdf]

Supplementary information

SYNTHESIS OF HIGHLY CONCENTRATED SILVER NANOPARTICLE DISPERSIONS AND THEIR INTEGRATION IN CELLULOSE FILMS FOR CATALYTIC AND ANTIBACTERIAL APPLICATIONS

Rodolfo P. P. Nunes,[1] Vitor G. Vital,[1] Nubia G. dos Santos[1], Suzan Pantaroto de Vasconcellos,[1] Larissa V. F. Oliveira,[2] Fernanda F. Camilo[1*]

[1]Chemistry Department, Institute of Environmental, Chemical and Pharmaceutical Sciences, Federal University of São Paulo, SP-09913-030, Diadema, Brazil

[2]Center of Natural Sciences and Humanities, Federal University of ABC, SP-09210-580, Santo André, Brazil

* *Corresponding author*: Fernanda Ferraz Camilo

Laboratório de Materiais Híbridos, Instituto de Ciências Ambientais, Químicas e Farmacêuticas, Universidade Federal de São Paulo.

Rua São Nicolau, 210, CEP: 09913-030, Diadema – SP, Brasil

E-mail: ffcamilo@unifesp.br

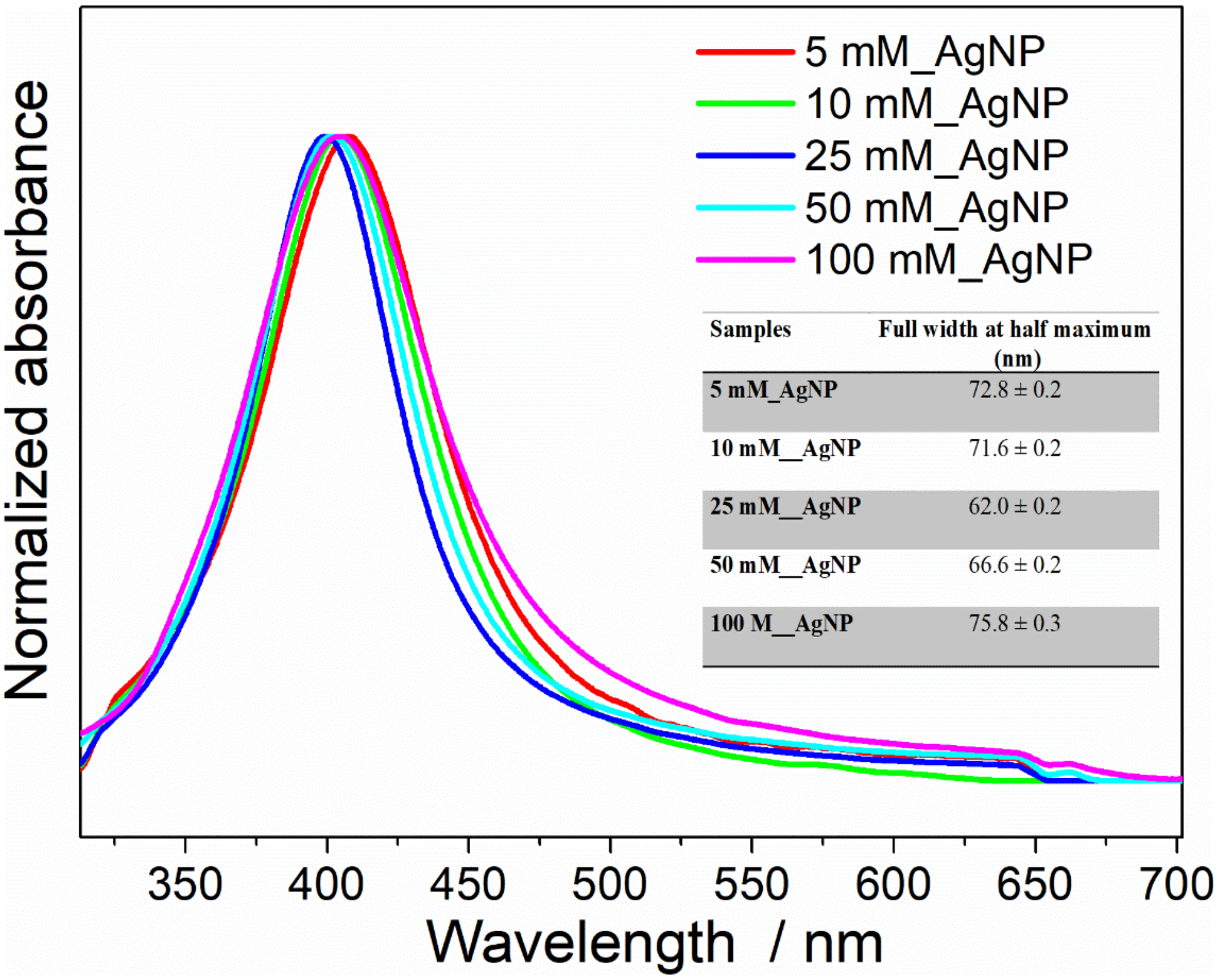

Figure S1. Normalized absorption spectra of silver nanoparticles prepared at different concentrations (5–100 mM). The inset shows the full width at half maximum (nm) for each sample.

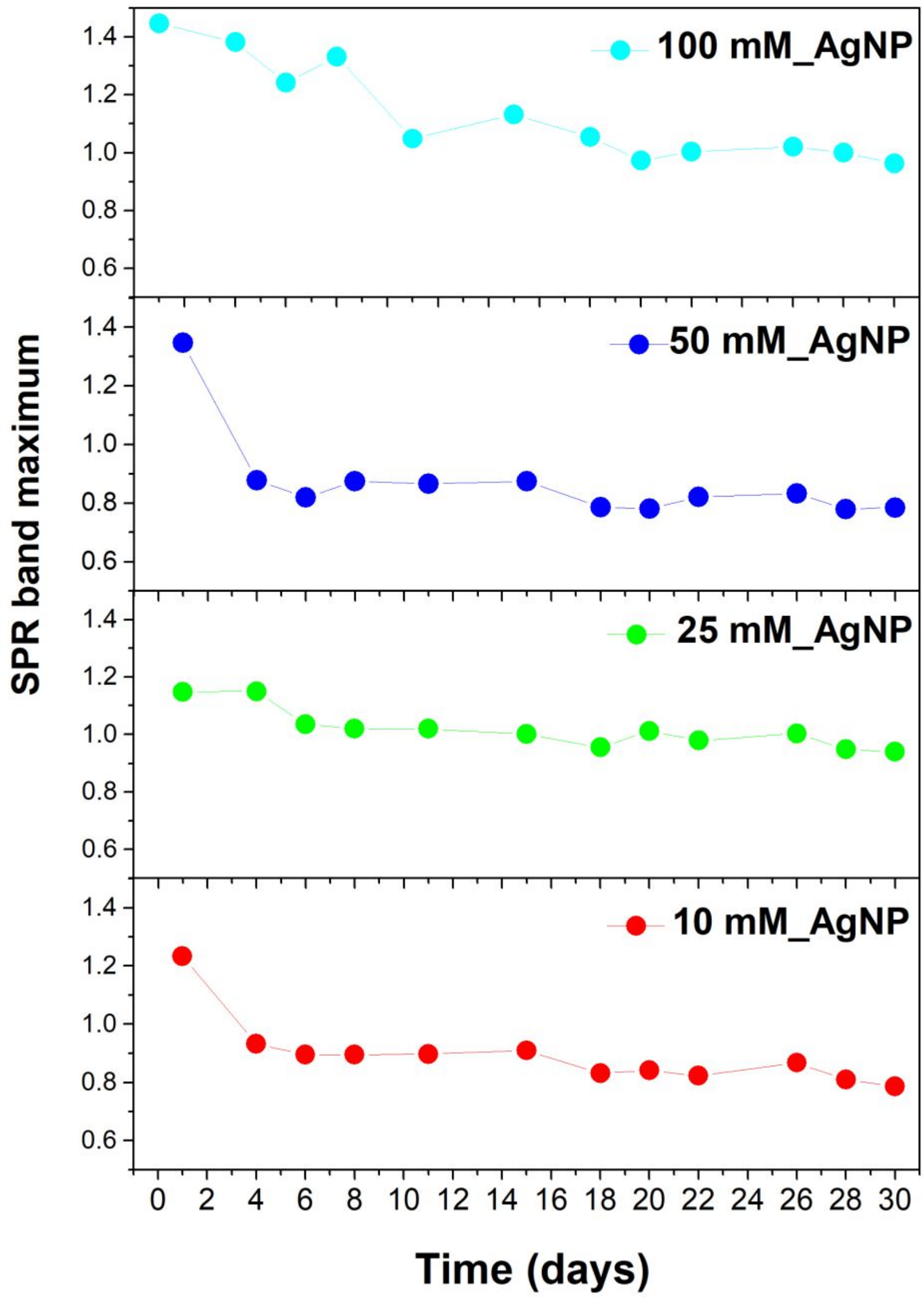

Figure S2. Temporal evolution of the surface plasmon resonance (SPR) band maximum of AgNP dispersions over 30 days. Minor fluctuations in absorbance were observed, with overall stability maintained throughout the period evaluated.

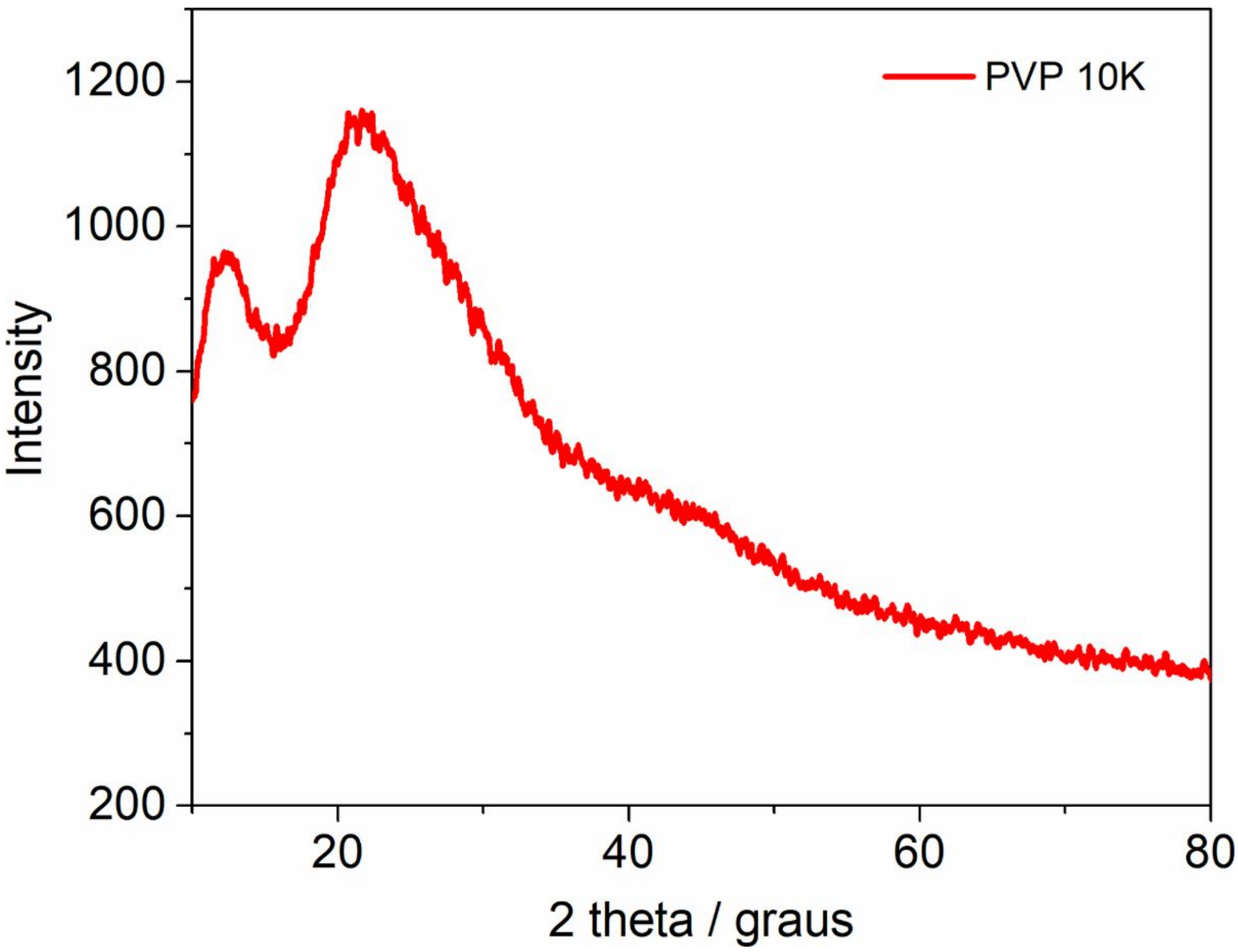

Figure S3. XRD pattern of pure PVP powder (Mw = 10 000 g·mol^{-1}, Aldrich).

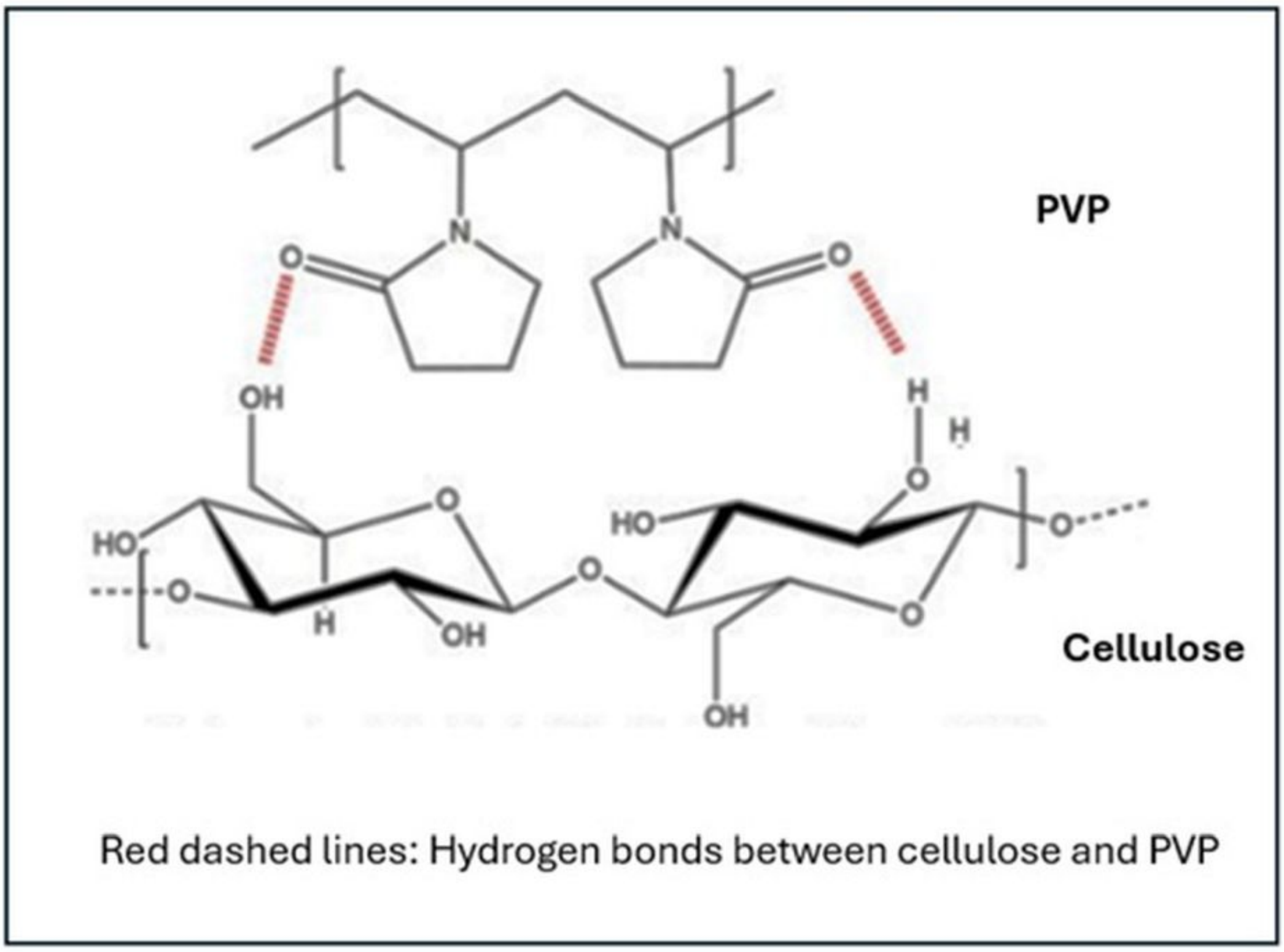

Figure S4. Schematic representation of hydrogen bonds (red dashed lines) between cellulose and PVP.

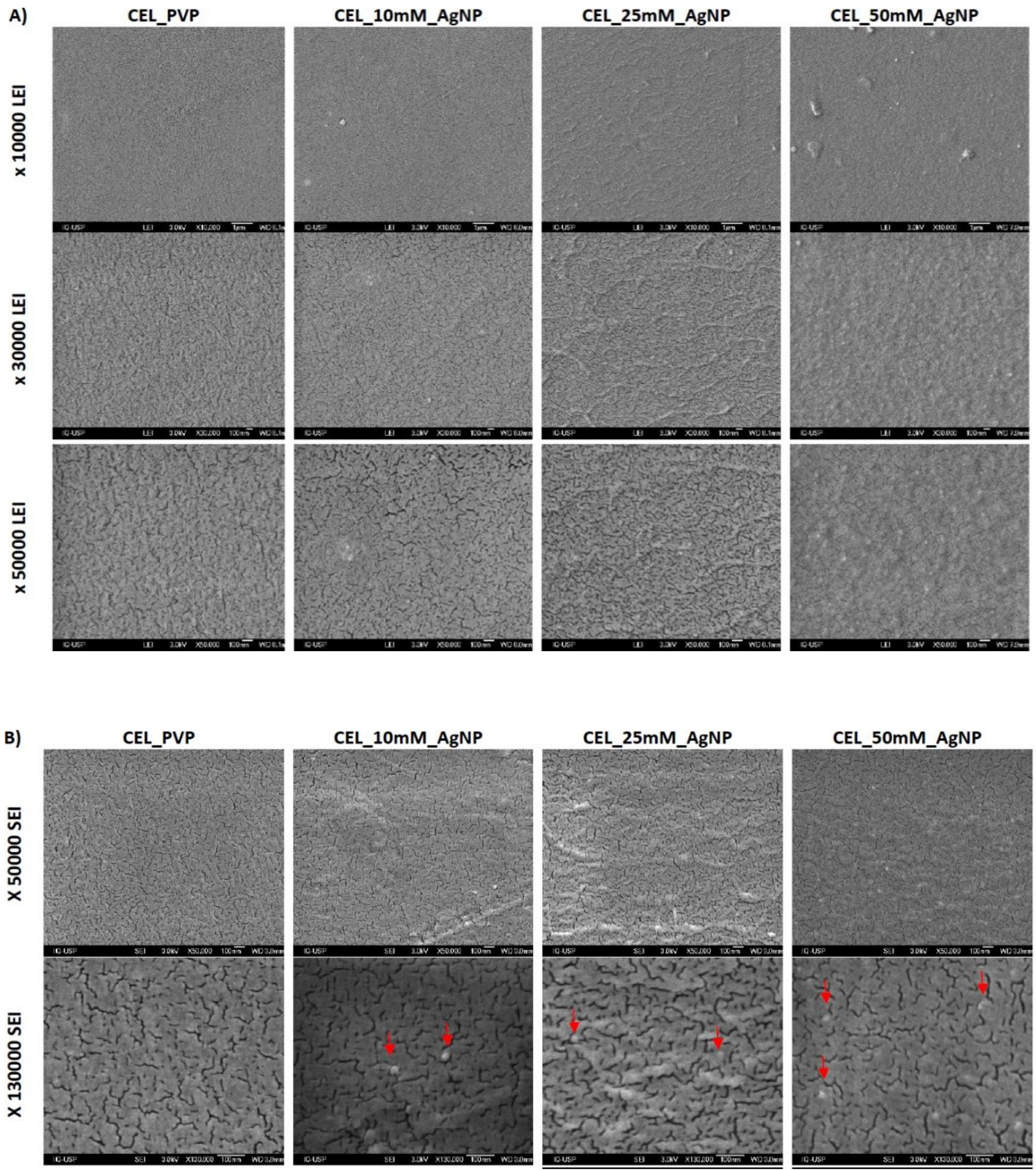

Figure S5. SEM micrographs of (A) CEL_PVP and CEL_AgNP films at different magnifications (×10 000, ×30 000, and ×50 000, LEI mode) and (B) selected regions acquired in SEI mode (×50 000 and ×130 000). Red arrows indicate the presence of AgNPs on the film surface.

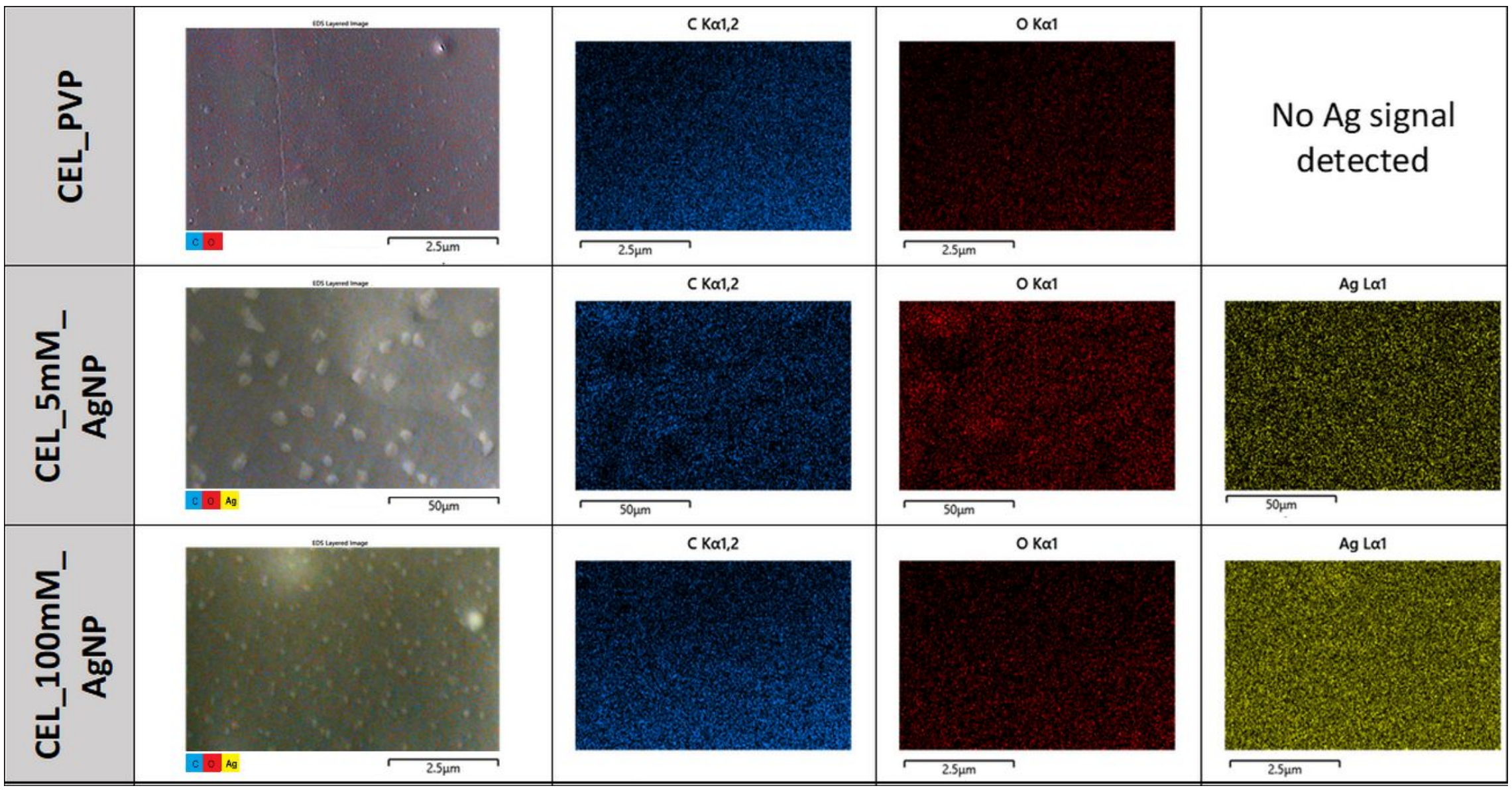

Figure S6. EDS elemental mapping analyses of CEL_PVP, CEL_5mM_AgNP, and CEL_100mM_AgNP films. For each sample, the overlaid SEM/EDS image and the corresponding elemental maps for C, O, and Ag are shown. The reference CEL_PVP film exhibits only C and O signals, with no detectable Ag signal. In contrast, both AgNP-impregnated films show detectable Ag throughout the analyzed regions, confirming the presence of silver species after impregnation. The Ag map is more intense for CEL_100mM_AgNP, consistent with the higher silver incorporation observed by ICP-OES.

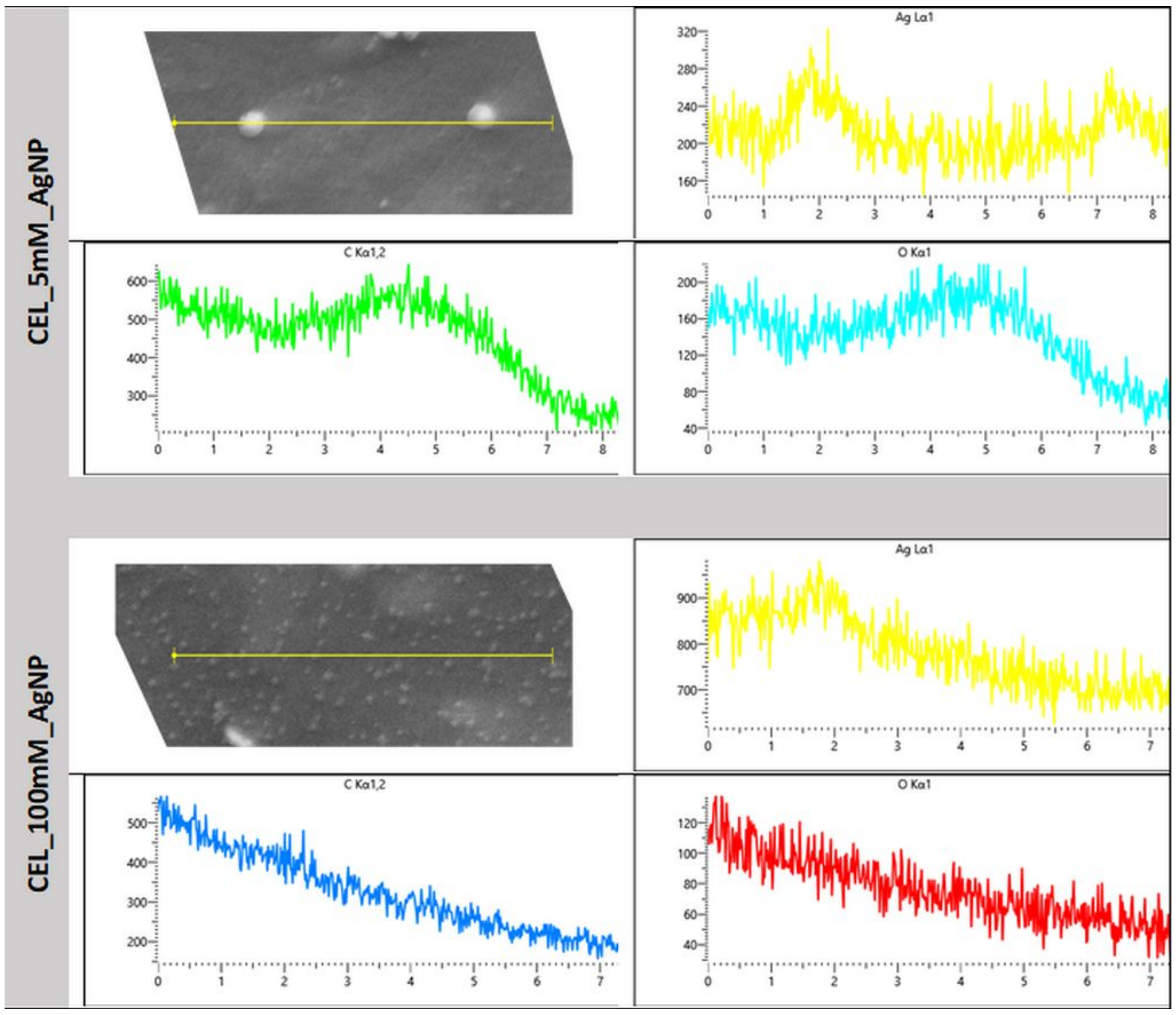

Figure S7. EDS line-scan analyses of CEL_5mM_AgNP and CEL_100mM_AgNP films. For each sample, the scanned path is indicated on the SEM image, and the corresponding intensity profiles for Ag, C, and O are shown. Detectable Ag signals were observed along the analyzed regions for both Ag-impregnated films, with local intensity variations along the scan path. The overall Ag signal was more intense for CEL_100mM_AgNP, consistent with the higher silver content determined by ICP-OES.

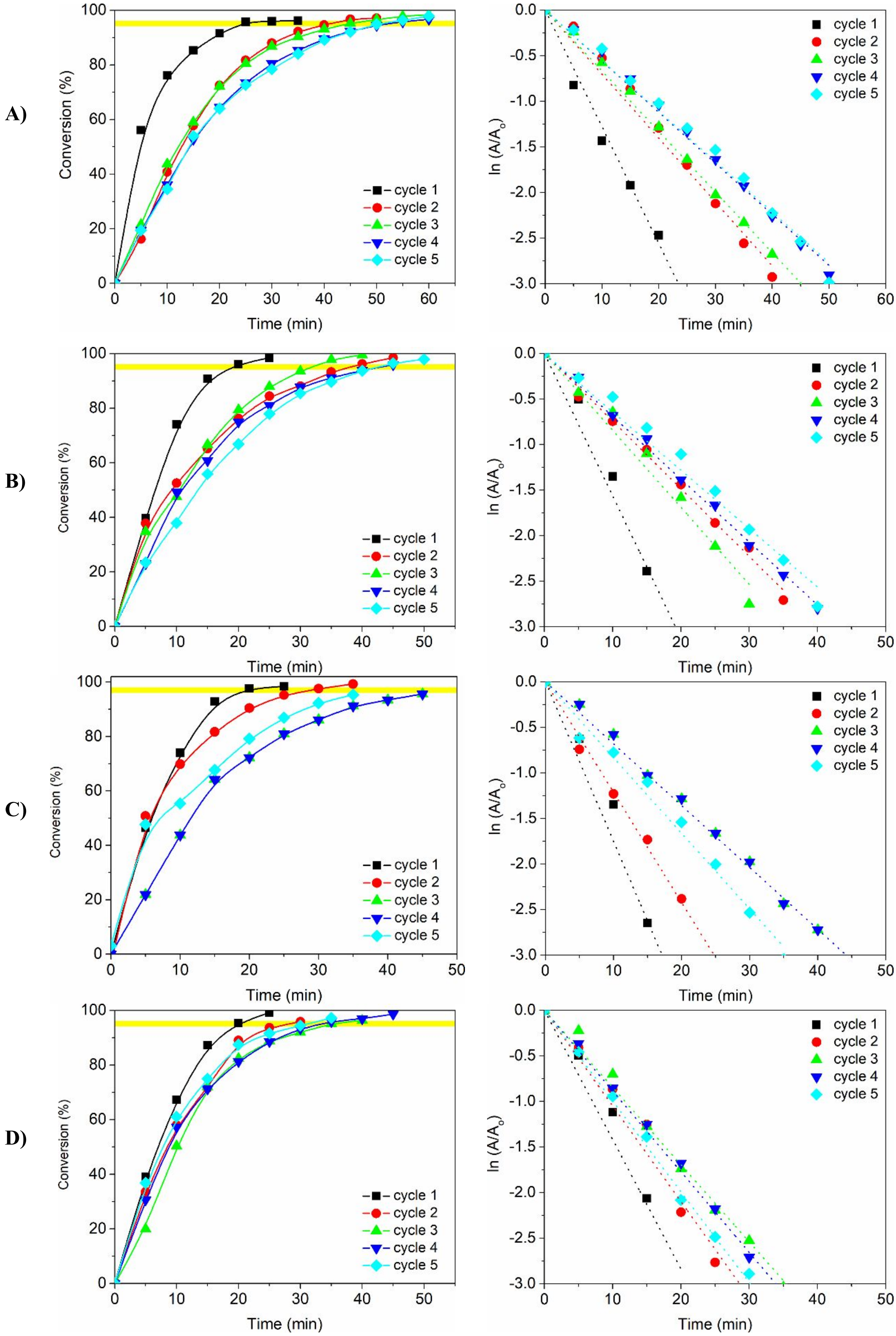

Figure S8. Kinetic curves of the catalytic reduction reaction obtained for (A) CEL_10mM_AgNP, (B) CEL_25mM_AgNP, (C) CEL_50mM_AgNP, and (D)

CEL_100mM_AgNP films. The plots show (left) conversion (%) versus time and (right) the corresponding first-order linear fitting [ln(A/A_0) versus time].

Table S1. Weight loss (%) of cellulose-based films at different temperature ranges and corresponding residue at 800 °C.

Sample	30-100 °C	200-380 °C	380-650 °C	Residue (%)
		Weight loss (%)		
**CEL**	5	61	29	0
**CEL_PVP**	14	31	54	0
**CEL_5mM_AgNP**	10	31	55.3	3.7
**CEL_10mM_AgNP**	9	39	45.5	6.5
**CEL_25mM_AgNP**	12	32	51.5	4.5
**CEL_50mM_AgNP**	9	34	49.5	7.5
**CEL_100mM_AgNP**	12	34	46.1	7.9
